# Integrative analysis of m7G methylation-associated genes prognostic signature with immunotherapy and identification of LARP1 as a key oncogene in head and neck squamous cell carcinoma

**DOI:** 10.3389/fimmu.2025.1520070

**Published:** 2025-02-13

**Authors:** Juan Xu, Zihao You, Zhongping Zhu, Min Liu, Zheng Zhang, Panpan Xu, Juanjuan Dong, Yuting Huang, Chao Wang, Haotian Qin

**Affiliations:** ^1^ Department of Oncology, Chaohu Hospital of Anhui Medical University, Hefei, China; ^2^ Anhui Medical University, Hefei, China; ^3^ Emergency Department, Peking University Shenzhen Hospital, Shenzhen, China; ^4^ Stomatological Center, Peking University Shenzhen Hospital, Shenzhen, China; ^5^ Department of Otolaryngology Head and Neck Surgery, Chaohu Hospital of Anhui Medical University, Hefei, China; ^6^ National & Local Joint Engineering Research Center of Orthopaedic Biomaterials, Peking University Shenzhen Hospital, Shenzhen, China; ^7^ Shenzhen Key Laboratory of Orthopaedic Diseases and Biomaterials Research, Peking University Shenzhen Hospital, Shenzhen, China

**Keywords:** N7-methylguanosine, head and neck squamous cell carcinoma, prognostic signature, immunotherapy response, ceRNA regulatory network, drug sensitivity

## Abstract

**Background:**

N7-methylguanosine (m7G) methylation is an RNA modification associated with cancer progression, but its specific role in head and neck squamous cell carcinoma (HNSCC) remains unclear.

**Methods:**

This study analyzed the differential expression of m7G-related genes (m7GRGs) in HNSCC using the TCGA-HNSCC dataset, identifying key pathways associated with the cell cycle, DNA replication, and focal adhesion. A LASSO-Cox regression model was constructed based on four m7GRGs (EIF3D, EIF1, LARP1, and METTL1) and validated with GEO datasets and clinical samples. Further validation of gene upregulation in HNSCC tissues was conducted using RT-qPCR and immunohistochemistry, while the role of LARP1 in HNSCC cells was assessed via knockout experiments.

**Results:**

The constructed model demonstrated strong predictive performance, with the risk score significantly correlating with prognosis, immune infiltration, and drug sensitivity. An external dataset and clinical specimens further confirmed the model’s predictive accuracy for immunotherapy response. Additionally, two regulatory axes—LINC00707/hsa-miR-30b-5p/LARP1 and SNHG16/hsa-miR-30b-5p/LARP1—were identified. LARP1 knockout experiments revealed that suppressing LARP1 markedly inhibited HNSCC cell proliferation, migration, and invasion.

**Conclusion:**

The m7GRG-based prognostic model developed in this study holds strong clinical potential for predicting prognosis and therapeutic responses in HNSCC. The identification of LARP1 and its related regulatory pathways offers new avenues for targeted therapy in HNSCC.

## Introduction

Head and Neck Squamous Cell Carcinoma (HNSCC) ranks as the sixth most common malignancy worldwide, with over 500,000 new cases and more than 140,000 deaths annually (1, 2). HNSCC is characterized by aggressive growth, distant metastasis, high postoperative recurrence, and poor prognosis. The main treatment modalities include surgery combined with radiotherapy, chemotherapy, and biological therapy (3, 4). Despite recent advances in therapeutic techniques, the overall prognosis for patients with advanced HNSCC has not significantly improved (5, 6). Therefore, there is an urgent need to identify new reliable clinical biomarkers to enhance the treatment efficacy and survival rates of HNSCC patients.

RNA methylation, a crucial post-transcriptional modification, regulates gene expression by influencing mRNA stability, translation efficiency, and degradation rate (7). In malignancies, this regulatory role can lead to the aberrant activation of oncogenes or inactivation of tumor suppressor genes, thereby promoting tumorigenesis and progression (8–10). RNA methylation modifications include 5-methylcytosine (m5C), N1-methyladenosine (m1A), N6-methyladenosine (m6A), and N7-methylguanosine (m7G) (11). N7-methylguanosine (m7G) modification is catalyzed by methyltransferase-like protein-1 (METTL1) and WD repeat domain 4 (WDR4), and these key enzymes exhibit abnormal activity in various malignancies. Studies have shown that m7G modification is associated with drug resistance and malignant behavior in multiple cancers, including glioma, liver cancer, and lung cancer (12). For instance, in glioma, the activity of O6-methylguanine-DNA methyltransferase (MGMT) is positively correlated with resistance to temozolomide (TMZ), highlighting the potential role of methylation modifications in tumor drug resistance (13). However, the specific role and mechanisms of m7G modification in HNSCC remain underexplored.

The tumor microenvironment (TME) is a critical factor in tumorigenesis and progression. RNA methylation also influences the infiltration and function of immune cells in the TME by regulating the expression of immune-related genes, thereby promoting immune evasion of tumor cells (14). Immune checkpoint inhibitors (ICIs) have been widely applied in the treatment of various malignancies, including non-small cell lung cancer, melanoma, renal cell carcinoma, and HNSCC (15). As research progresses, more tumor types are found to benefit from ICI therapy. However, a comprehensive and in-depth study of the role of m7G modification in HNSCC and its relationship with immunotherapy response is still lacking.

In this study, we analyzed m7G methylation-related genes (m7GRGs) in HNSCC using bioinformatics methods. We validated the expression of m7GRGs in clinical tissues and three HNSCC cell lines via RT-qPCR. The relationship between m7GRGs and HNSCC prognosis was explored, leading to the development of a prognostic model based on four m7GRGs and the construction of a ceRNA regulatory network. La-related protein 1 (LARP1) was identified as a key oncogene. Knockout experiments in two HNSCC cell lines demonstrated that LARP1 knockout significantly inhibited cell proliferation, migration, and invasion. Our findings offer new insights into the treatment and prognostic assessment of HNSCC.

## Materials and methods

### Data source and preprocessing

RNA sequencing data and clinical information for 504 HNSCC cases were obtained from TCGA (https://portal.gdc.cancer.gov//) (16). Data were normalized to Transcripts Per Million (TPM) and visualized using R software (v4.0.3) with the “ggplot2” package. Based on published data (9, 17, 18), 45 genes related to m7G were identified.

### Clinical data and tissue sample collection

Clinical data and tissue samples were collected from Chaohu Hospital of Anhui Medical University and Peking University Shenzhen Hospital, involving 76 HNSCC patients admitted between September 2016 and September 2018. Formalin-fixed, paraffin-embedded HNSCC tissues and adjacent normal tissues (0.5 cm) were obtained with complete clinical data and follow-up information. The study was approved by the Ethics Committees of both hospitals (No. KYXM202310004 and 2022-117) and adhered to the Helsinki Declaration (2013 revision). Written informed consent was obtained from all patients.

### Subtype establishment

Consensus clustering of the 45 m7GRGs from the TCGA expression matrix was performed using the R package ConsensusClusterPlus (v1.54.0) (19), with k set to a maximum of 6, drawing 80% of the total sample 100 times. Optimal classification was evaluated by varying cluster numbers (k=2-6), using the consensus matrix and cumulative distribution function (CDF). Clustering heatmaps were analyzed with the R package pheatmap (v1.0.12), retaing motifs with variance > 0.1. Based on the expression profiles of m7GRGs, TCGA cases were divided into two clusters: Cluster 1 (N = 207) and Cluster 2 (N = 297) subtypes.

### Identification and enrichment analysis of differentially expressed genes

The R package Limma (v3.40.2) (20) identified differentially expressed genes (DEGs) between molecular subtypes. Significant mRNA differential expression thresholds were set at “Adjusted P <0.05 and |log2 (fold change)| > 1”. Gene Ontology (GO) and KEGG pathway enrichment were performed using the R package clusterProfiler (v3.18.0) (21). Gene Set Enrichment Analysis (GSEA) (22) identified potential biological pathways, analyzed with the R package GSVA (23), using method = ‘ssgsea’. The STRING database (https://string-db.org/) (24) (version 11.5) analyzed the protein-protein interaction (PPI) network of m7GRGs.

### Genetic variation

The CNV module of the Gene Set Cancer Analysis (GSCA) (http://bioinfo.life.hust.edu.cn/GSCA) analyzed amplification/deletion and heterozygous/homozygous of m7GRGs in HNSCC and correlated m7GRG expression with CNV. 509 HNSCC patients somatic mutations were visualized using the maftools package (25), covering six mutation types. The impact of m7GRGs on HNSCC patient survival was assessed using 523 HNSCC samples from the cBioPortal (http://www.cbioportal.org/) (26).

### Relationship between m7GRGs and HNSCC clinical pathological characteristics and prognosis

Clinical pathological data for 504 HNSCC patients from TCGA (**Supplementary Table S1**) were included, encompassing variables such as sex, tumor stage, grade, and smoking status. Significant P-values were further analyzed using chi-square tests and represented as -log10 (P-value). The correlation between m7GRG expression and clinical staging data was verified using UALCAN (http://ualcan.path.uab.edu/index.html) (27). Protein expression of m7GRGs was obtained from CPTAC (https://cptac-dataportal.georgetown.edu) (28), and the Human Protein Atlas (HPA) (https://www.proteinatlas.org) (29) analyzed m7GRG protein levels in HNSCC tissues.

### Construction of m7GRG prognostic signature and predictive nomogram

Feature selection was conducted using LASSO regression with 10-fold cross-validation in the R package glmnet. After performing 10-fold cross-validation, the optimal tuning parameter, lambda, was identified. This minimal lambda value was chosen because it demonstrated the best performance on the validation dataset. Subsequently, the LASSO Cox regression model was fitted using this optimal lambda value. Cross-validation is a widely accepted method for assessing the generalization ability of predictive models by repeatedly training and evaluating the model on different subsets of the data. By selecting lambda through this rigorous cross-validation process, the robustness and reliability of the model selection were ensured. According to the results of multivariate Cox regression analysis, the prognostic m7GRG risk score was calculated as follows: Risk score = ∑i (Coefficient (mRNA_i) × Expression (mRNA_i)). Based on the mean risk score, TCGA-HNSCC patients were divided into low-risk and high-risk groups. Survival differences were assessed using Kaplan-Meier analysis, and model accuracy was evaluated with time-dependent receiver operating characteristic (timeROC) analysis. Furthermore, the validation cohort was used to verify the accuracy of the m7GRG signature based on the above formula. The TCGA-HNSCC dataset was randomly split into two validation sets: Validation Set 1 (n = 251) and Validation Set 2 (n = 252). In addition, the GEO database (https://www.ncbi.nlm.nih.gov/gds) (30) (including GSE65858, GSE41613, and GSE85446) was utilized as an external validation cohort to further corroborate the findings. The Optimal cutoffs were determined using the “surv_cutpoint” function in “survminer” R package. Kaplan-Meier and ROC curves validated the prognostic gene markers. Univariate and multivariate Cox regression analyses were visualized with forestplot. A nomogram predicting 1-, 3-, and 5-year OS, PFS, and DSS was constructed using the rms package. The calibration curve was employed to assess the consistency of the nomogram, and its validity was further confirmed through time-dependent ROC analysis, time-dependent AUC values, and decision curve analysis (DCA). These analyses were conducted to evaluate whether the nomogram demonstrates a stronger association with clinical net benefits compared to other models.

### Immune cell infiltration and immunotherapy response analysis

Immune cell infiltration levels and expression differences of immune checkpoint-related genes (HAVCR2, SIGLEC15, PDCD1, CD274, PDCD1LG2, TIGIT, CTLA4, and LAG3) were compared using six algorithms in the R package immunedeconv (31), including TIMER (32), xCell (33), MCP-counter (34), CIBERSORT (35), EPIC (36), and quantTIseq (37), analyzed via the Wilcoxon test. The potential immune checkpoint blockade response was predicted using the TIDE (Tumor Immune Dysfunction andExclusion) algorithm (38). The ESTIMATE algorithm (39) estimated Immune cell abundance (immune score), stromal cell infiltration level (stromal score), and the combined (ESTIMATEScore). Results were visualized using “ggplot2” and “pheatmap”. Immune cell abundance was analyzed using TIMER (https://cistrome.shinyapps.io/timer/) (40). Single-sample Gene Set Enrichment Analysis (ssGSEA) (via R packages GSVA) (23) quantified the infiltration levels of 24 immune cell types. The response of m7GRGs to immunotherapy was predicted using GSE91061, GSE135222, and GSE78220 datasets.

### TMB, MSI, mRNAsi and drug sensitivity analysis

Spearman correlation analysis between Tumor Mutation Burden (TMB), Microsatellite Instability (MSI), mRNA stemness index (mRNAsi) (41), and prognostic model risk score was visualized using ggstatsplot. Drug sensitivity and gene expression profile data from the GDSC (https://www.cancerrxgene.org/) (42) and CTRP (https://portals.broadinstitute.org/ctrp/) databases predicted chemotherapy response, achieved by the R package pRRophetic (43).

### Single-cell analysis

The t-SNE plot and heatmap of HNSCC_GSE103322 were presented using TISCH (http://tisch.comp-genomics.org/) (44). Correlation between prognostic m7GRGs expression levels and cancer-associated fibroblasts (CAFs) was plotted using TIMER2.0 (http://timer.cistrome.org/) (45).

### Correlation analysis with CRGs

CRGs analyzed included ATP7B, CDKN2A, LIPT1, DLAT, PDHA1, LIAS, PDHB, GLS, DLD and FDX1 (46). Spearman correlation analysis was performed between m7GRGs and CRGs expression in TCGA-HNSCC samples. The Wilcoxon test analyzed expression level differences of CRGs between high and low m7GRG expression groups, visualized using “ggplot2”.

### Prediction of potential miRNA and lncRNA target genes

Potential miRNA and lncRNA target genes were predicted using RNAInter (http://www.rnasociety.org/rnainter/) (47), ENCORI (http://starbase.sysu.edu.cn/) (48), and miRNet (http://www.mirnet.ca/) (49) databases. An mRNA-miRNA, miRNA-lncRNA regulatory network was constructed using Cytoscape (version 3.7.1; http://www.cytoscape.org/) (50). The correlation and prognostic value of predicted miRNA and lncRNA with m7GRGs in HNSCC were validated using ENCORI and Kaplan–Meier plotter databases.

### Cell lines and culture conditions

A normal squamous cell line (NOK) and three HNSCC cell lines (HN6, HSC3, SCC9) were obtained from ATCC (Manassas, VA, USA). HN6 and HSC3 cells were cultured in DMEM (Sigma, D5546) with 10% fetal bovine serum (FBS) (Gibco, 10099-141C) and 1% penicillin-streptomycin (PS) (Gibco, 15070063). SCC9 cells were cultured in DMEM with 10% FBS, 1% PS, and 1 ng/mL hydrocortisone (MCE, HY-N0583). NOK cells were maintained in Defined Keratinocyte-SFM (Gibco, 10744019) with growth supplement and 1% PS. All cultures were incubated at 37°C in a humidified incubator with 5% CO2. Cells were seeded in six-well plates for 24 hours and then transfected with shRNA-LARP1 (GeneRulor, Zhuhai) using Lipofectamine 3000 (Invitrogen, USA) at 60-70% confluency. RNA was extracted 48 hours post-transfection to assess transfection efficiency. Experiments were performed in triplicate.

### Proliferation and colony formation assays

For proliferation assays, 2000 cells were seeded into 96-well plates. Cell viability was assessed daily for five days using the Cell Counting Kit-8 (CCK-8) assay (Dojindo, Japan) according to the manufacturer’s instructions; each experiment was performed in triplicate. For colony formation assays, 1000 cells were seeded into six-well plates and cultured for approximately two weeks. Visible colonies were fixed with 4% paraformaldehyde, stained with 1% crystal violet, and counted.

### Wound healing assay

Cells were seeded in six-well plates and grown to 90% confluence. A wound was created using a pipette tip, and detached cells were removed with PBS. Images of the wound area were captured 24 hours post-wounding, and the wound area was measured using ImageJ.

### Transwell assay

Cell migration and invasion were analyzed using 24-well Transwell chambers with or without Matrigel coating (Corning, NY, USA, 354480, 3422). Cells suspended in serum-free medium were placed in the upper chamber, while medium containing 10% FBS was added to the lower chamber. After 24 hours of incubation, non-migrated cells in the upper chamber were removed. Migrated cells on the Transwell membrane were fixed with methanol, stained with crystal violet, and counted under a microscope (×100 magnification) in five randomly selected fields.

### RNA extraction and RT-qPCR

Total RNA was extracted using the Quick-RNA MiniPrep Kit (Zymo Research, R1054). Target gene expression was detected using the miScript SYBR Green PCR Kit (Qiagen, Germany) on a LightCycler 96 real-time PCR system (Roche Diagnostics GmbH, Mannheim, Germany). Relative mRNA expression levels were quantified using the 2-^△△CT^ method, with GAPDH as the reference gene. PCR primers are listed in **Supplementary Table S2**.

### Validation of m7GRG protein expression by immunohistochemistry

DRG protein expression in HNSCC tissues was evaluated by immunohistochemistry (IHC). Formalin-fixed, paraffin-embedded tissue samples were sectioned at 4 µm, deparaffinized, rehydrated, and antigen retrieval was performed in EDTA. Endogenous peroxidase activity was blocked with 3% hydrogen peroxide. Non-specific binding was reduced with 10% normal goat serum. Rabbit monoclonal antibodies against m7GRG (ab155419, ab172623, ab86359, ab271063; 1:500, Abcam, UK) were used as primary antibodies, and samples were incubated at room temperature for 1 hour. After washing with PBS, biotin-labeled secondary antibodies and streptavidin-biotin complex-horseradish peroxidase were added sequentially, with each incubation lasting 10 minutes at room temperature. Samples were then stained with DAB, dehydrated, and mounted with resin. The results were quantified using ImageJ software.

### Statistical analysis

All experiments were repeated at least three times independently. The quantitative data are presented as mean + standard deviation (SD). SPSS Statistics 24.0 (IBM Corp Armonk, NY, USA) was utilized to analyze the data. Student’s t-test was used to compare the differences between two groups, and one-way analysis of variance(ANOVA) was used to compare the difference between three or more groups. A two-tailed p-value < 0.05 was considered statistically significant. In the Figures, asterisks indicate the p-value: *p< 0.05, **p<0.01, and ***p< 0.001.

## Results

### Identification and analysis of m7GRGs clustering in HNSCC

**Figure 1:**
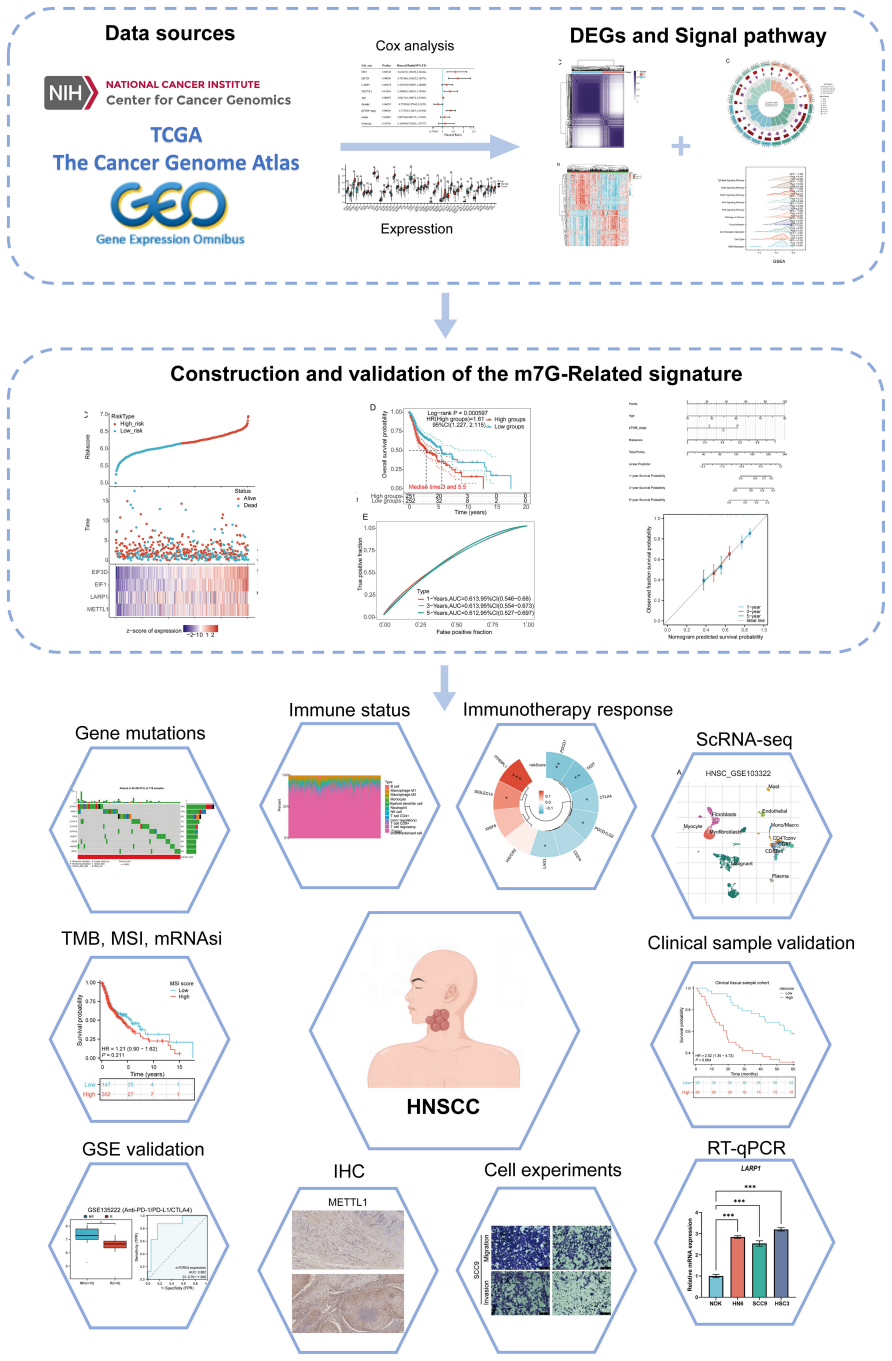
Flowchart of the present study.

**Figure 2:**
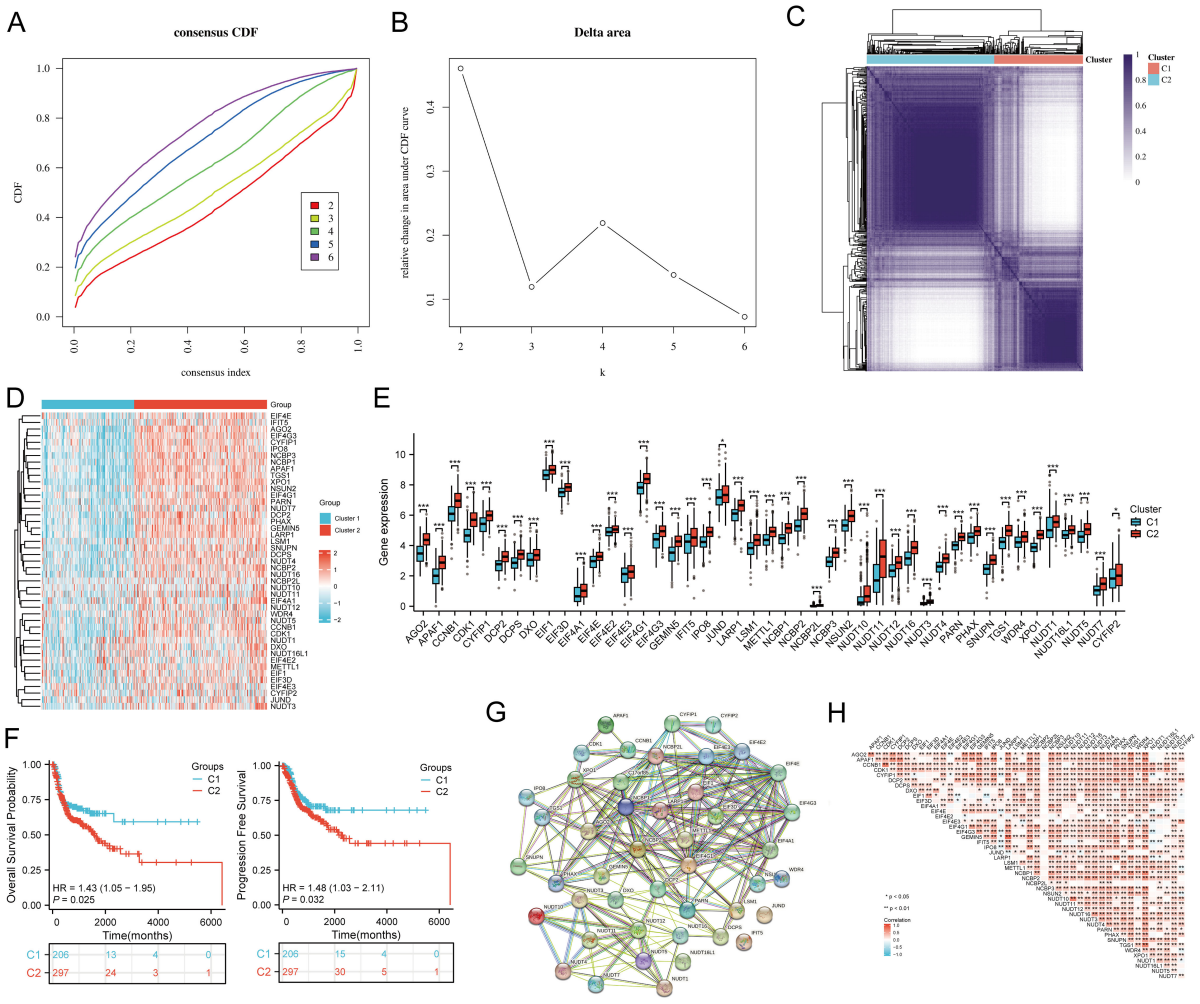
Identification of common clusters based on the expression of m7G-related genes (m7GRGs). **(A)** Cumulative distribution function (CDF) for k = 2–6; **(B)** Relative change in area under the CDF curve (CDF Delta area) for k = 2–6; **(C)** Consensus clustering matrix for k = 2; **(D)** Heat map of m7GRG expression in different subtypes, with red indicating high expression and blue indicating low expression; **(E)** Expression levels of 45 m7GRGs in cluster 1 and cluster 2, including the quartile ranges of upper and lower representative values of the box, with the line in the box representing the median value; **(F)** Kaplan-Meier survival analysis was conducted according to two clusters. **(G)** Protein-protein interaction network (PPI) of m7GRGs (STRING) ; **(H)** Pearson's correlation analysis of 45 m7GRG expressions in HNSCC. *p<0.05, **p<0.01, ***p<0.001.

The study workflow is illustrated in **Figure 1**. Based on the expression levels of 45 m7GRGs in HNSCC, we performed consensus clustering on 504 HNSCC samples from the TCGA database. The tumor samples were divided into k (k = 2-6) distinct clusters. After analyzing the clustering results, we selected k = 2, which accurately grouped the HNSCC patients into two clusters: C1 (N = 207) and C2 (N = 297) (**Figures 2A–D**). Compared to Cluster 1, all m7GRGs were upregulated in Cluster 2 (**Figure 2E**). Kaplan-Meier survival analysis revealed that the overall survival (OS) and progression-free survival (PFS) of C2 patients were significantly lower than those of C1 patients (**Figure 2F**). The protein-protein interaction (PPI) network of the 45 m7GRGs was constructed using the STRING tool (**Figure 2G**). Further statistical analysis of the interaction strengths between the genes identified key hub genes, including LARP1, EIF4E, NCBP2, NCBP1, EIF4E2, and EIF4E3 (**Figure 2H**).

### Differentially expressed genes and functional enrichment analysis

**Figure 3:**
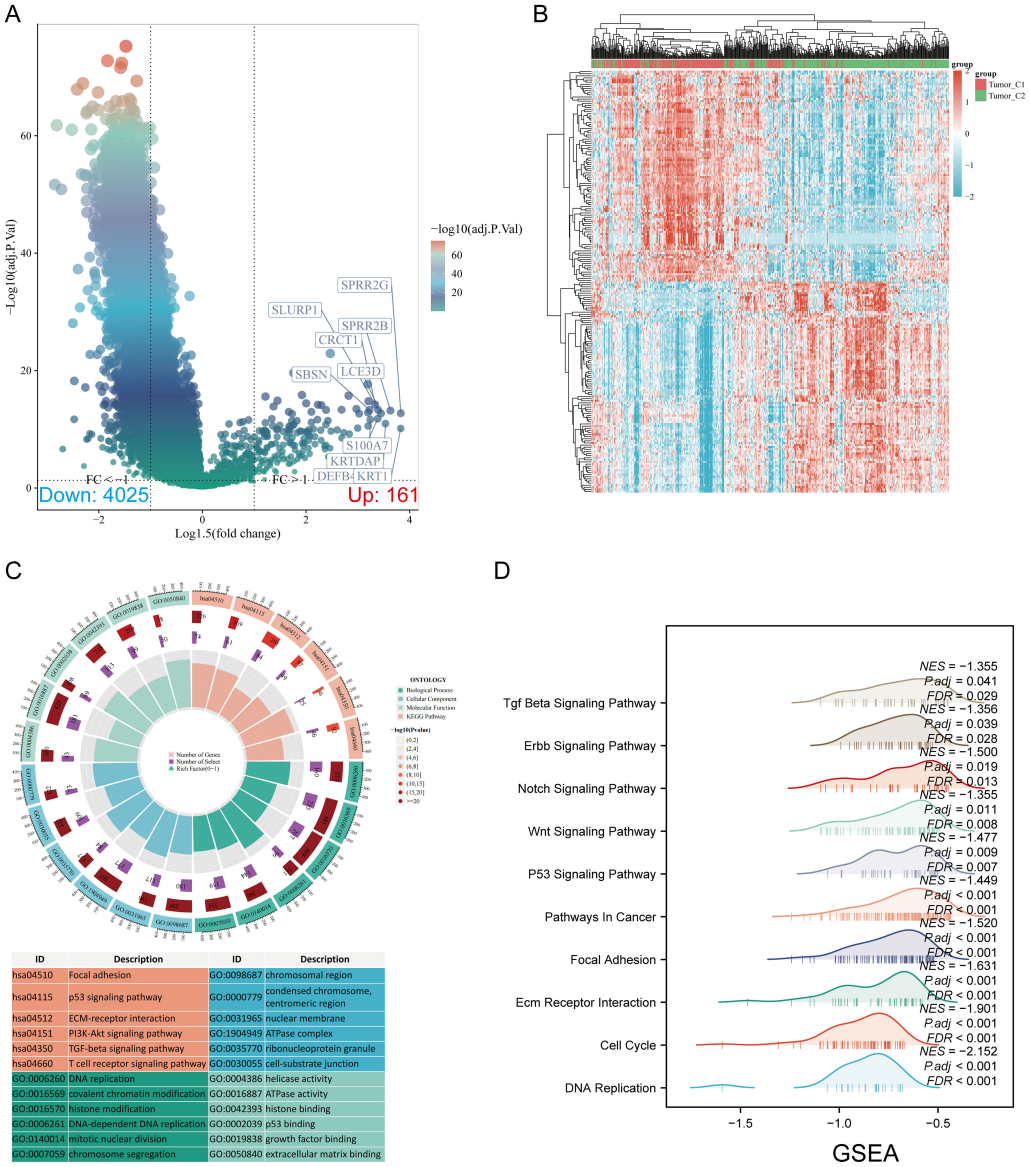
Screening of differentially expressed genes (DEGs) between m7GRG subtypes and functional enrichment analysis of DEGs. **(A)** Volcano plot of DEGs between C1 and C2 subtypes, constructed using fold change and adjusted p-values. The left side represents significantly downregulated genes, while the right side represents significantly upregulated genes. **(B)** Heat map of DEGs between C1 and C2 subtypes; **(C)** KEGG and GO enrichment analysis results for DEGs; **(D)** Enrichment map from GSEA.

Volcano and heat maps were constructed based on DEGs between the C1 and C2 subtypes (**Figures 3A, B**). The DEGs identified between C1 and C2 subtypes included 161 upregulated genes and 4025 downregulated genes. KEGG enrichment analysis highlighted pathways such as focal adhesion, p53 signaling, ECM-receptor interaction, PI3K-Akt signaling, TGF-beta signaling, and T-cell receptor signaling. GO analysis revealed enrichment in DNA replication, covalent chromatin modification, chromosomal region, cell-matrix adhesion, ATPase activity, and extracellular matrix binding (**Figure 3C**). GSEA indicated significant enrichment in cell cycle, focal adhesion, adherens junction, ECM-receptor interaction (**Figure 3D**; **Supplementary Table S3**).

### Genetic variation analysis

**Figure 4:**
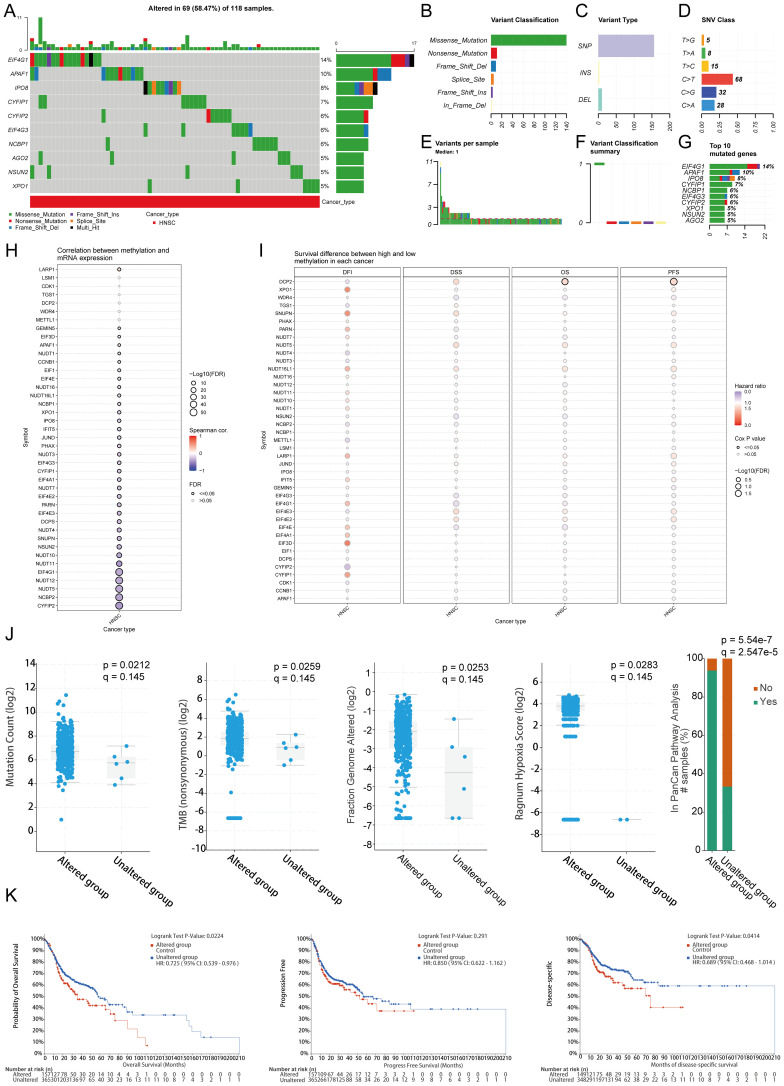
Correlation analysis of genetic alterations in m7GRGs. **(A)** Distribution of mutation types in the top 10 m7GRGs in HNSCC; **(B–D)** Variant classification, variant type, and SNV class; **(E)** Mutation burden per sample; **(F)** Summary of variant classification; **(G)** Top 10 mutated genes in HNSCC. SNP, single nucleotide polymorphism. **(H)** Relationship between methylation levels and m7GRG expression; **(I)** Correlation between methylation levels and survival rates in HNSCC patients; **(J)** Association between mutation count, TMB (nonsynonymous), fraction genome altered, Ragnum hypoxia score, PanCan pathway analysis, and m7GRG alterations in HNSCC tissues; **(K)** Association between m7GRG alterations and shorter OS, PFS, and DSS in HNSCC patients.

Using the GSCA database, an Oncoplot revealed the top 10 genes with SNVs among m7GRGs, with EIF4G1 (14%) and APAF1 (10%) having the highest mutation frequencies (**Figure 4A**). Missense mutations were the most common mutation type (**Figure 4B**). Single nucleotide polymorphisms (SNPs) were more frequent than insertions and deletions (**Figure 4C**), with C>T being the most common SNV type (**Figure 4D**). Analysis of the number of base changes per patient showed a median and maximum of 1 and 11 mutations, respectively (**Figure 4E**). A boxplot of each variant classification showed the frequency of occurrences (**Figure 4F**). Recalculating the top 10 mutated genes, considering multiple hits, yielded slightly different results (**Figure 4G**). There was a significant negative correlation between mRNA expression of m7GRGs and gene methylation levels (**Figure 4H**). Lower methylation levels were significantly associated with poorer prognosis in HNSCC patients (**Figure 4I**). Through the GSCA database, analysis of the CNV landscape of 45 m7GRGs in HNSCC showed high rates of heterozygous deletion/amplification (**Supplementary Figure S1A**). NCBP2, LSM1, PHAX, NSUN2, and NCBP3 had higher CNV rates compared to other genes (**Supplementary Figure S1B**). CNV analysis indicated both heterozygous amplification and heterozygous deletion of m7GRGs (**Supplementary Figure S1C**). Detailed mutation sites of EIF3D, EIF1, LARP1, and METTL1 included missense mutations, splice sites, nonsense mutations, and frameshift deletions (**Supplementary Figure S2A**). **Supplementary Figure S2B** provides a detailed distribution ratio of CNVs in m7GRGs in HNSCC. Subsequent analysis of HNSCC samples from the cBioPortal database revealed significant differences between the mutation group and the non-mutation group in mutation count (p = 0.0212, q = 0.145), TMB (nonsynonymous) (p = 0.0259, q = 0.145), genome alteration fraction (p = 0.0253, q = 0.145), and Ragnum hypoxia score (p = 0.0283, q = 0.145), especially in the PanCan Pathway Analysis (p = 5.54e-7, q = 2.547e-5) (**Figure 4J**). Survival analysis indicated that genetic alterations in m7GRGs were significantly associated with shorter OS (p = 0.0224, HR = 0.725 [0.539 - 0.976]) and DSS (p = 0.0414, HR = 0.689 [0.468 - 1.0144]) but not with PFS (p = 0.291, HR = 0.850 [0.622 - 1.162]) (**Figure 4K**). These results suggest that alterations in m7GRGs impact the prognosis of HNSCC patients.

### Prognostic value analysis

**Figure 5:**
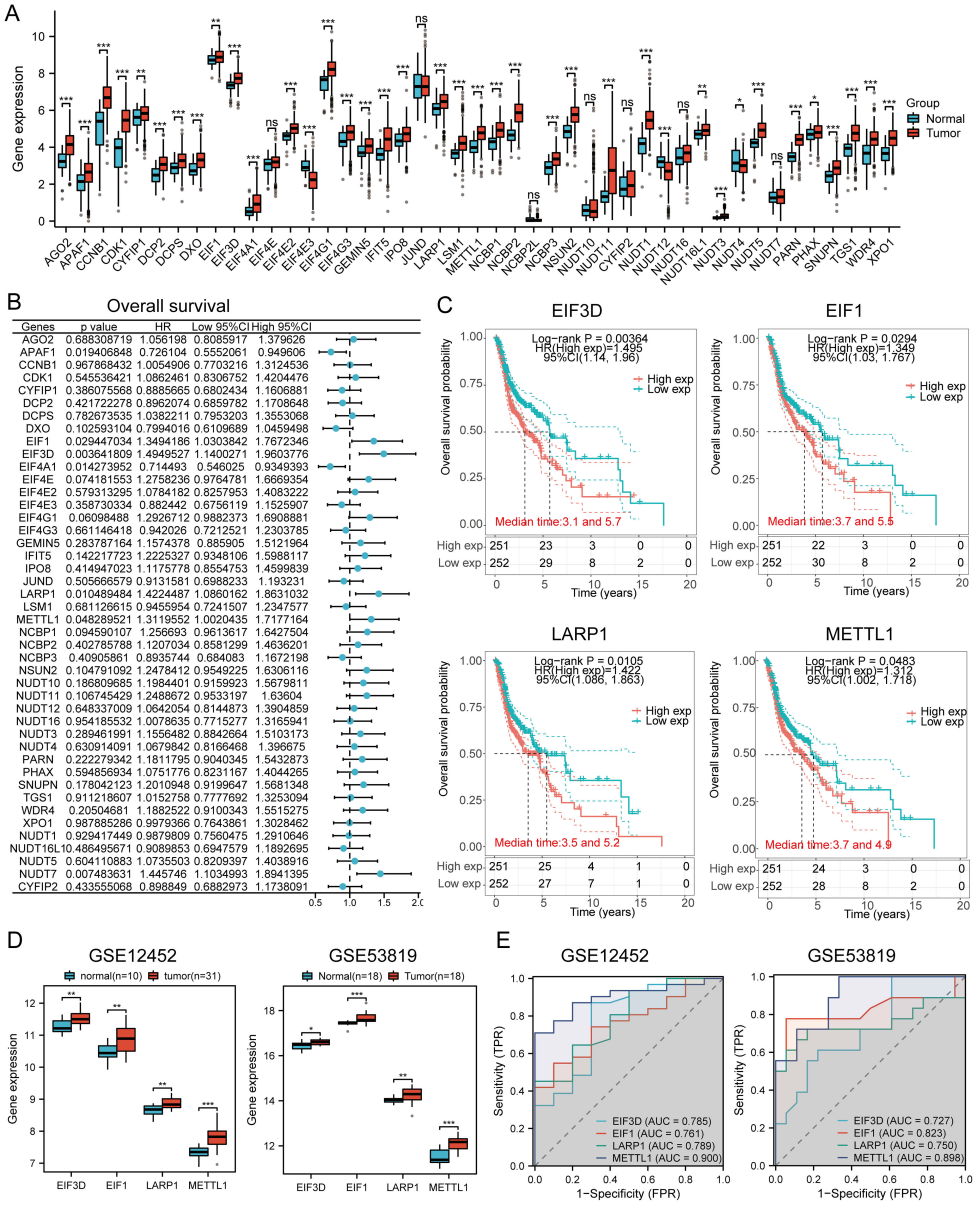
Prognostic value and gene expression. **(A)** Expression levels of 45 m7GRGs in HNSCC and adjacent tissues; **(B)** Univariate Cox regression analysis of m7GRGs; **(C)** Prognostic value of four m7GRGs (EIF3D, EIF1, LARP1, and METTL1) in high and low expression groups among HNSCC patients; **(D)** mRNA expression of prognostic m7GRGs in GSE12452 and GSE53819 datasets; **(E)** ROC curves evaluating the diagnostic ability of prognostic m7GRG expressions in GSE12452 and GSE53819 datasets. n.s. no significance (p > 0.05), *p<0.05, **p<0.01, ***p<0.001.

Compared to normal tissues, most genes were upregulated in cancer tissues, while EIF4E3, NUDT12, and NUDT4 were downregulated (**Figure 5A**). Forest plot analysis revealed that high expression of EIF3D, EIF1, LARP1, NUDT7, and METTL1 was associated with lower overall survival in HNSCC patients (**Figure 5B**). Specifically, high expression of EIF3D (p = 0.00364, HR = 1.495 [1.14 - 1.96]), EIF1 (p = 0.0294, HR = 1.349 [1.03 - 1.767]), LARP1 (p = 0.0105, HR = 1.422 [1.086 - 1.863]), and METTL1 (p = 0.0483, HR = 1.312 [1.002 - 1.718]) indicated poor prognosis (**Figure 5C**). The expression levels of prognostic m7GRGs were significantly upregulated in high expression groups in GSE12452 and GSE53819 datasets (**Figure 5D**). ROC curve analysis showed AUC values greater than 0.7, indicating high diagnostic accuracy of prognostic m7GRGs in HNSCC (**Figure 5E**).

### Pathological expression of m7G-related proteins in HNSCC

Compared to normal tissues, the protein expressions of EIF3D, EIF1, and METTL1 were significantly higher in HNSCC tissues according to the CPTAC database (**Supplementary Figure S3A**). Immunohistochemistry staining revealed moderate to high expression of prognostic m7GRGs in HNSCC tissues, while expression was lower in normal tissues. However, immunohistochemistry results for EIF1 were not available (**Supplementary Figure S3B**).

### Construction of prognostic model

**Figure 6:**
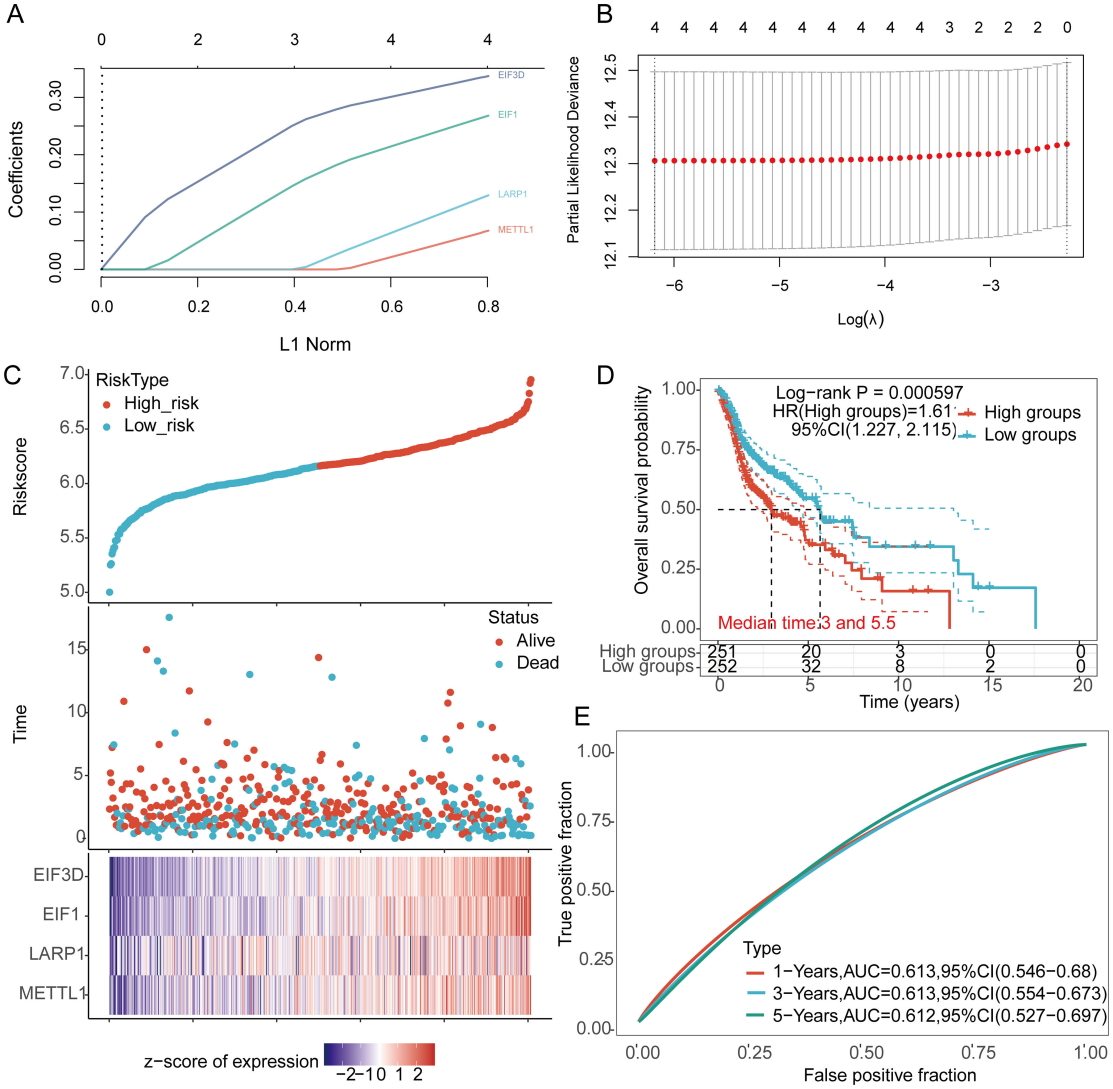
Construction of a prognostic model using m7GRGs in HNSCC tissue. **(A)** LASSO coefficient curve of four m7GRGs; **(B)** Ten-fold cross-validation error rates; **(C)** Distribution of risk score, survival status, and expression of prognostic m7GRGs in HNSCC patients; **(D)** Overall survival curve for high/low-risk groups of HNSCC patients; **(E)** Time-dependent ROC curve for 1-, 3-, and 5-year OS for m7GRGs.

LASSO Cox regression analysis was performed to construct a prognostic model based on EIF3D, EIF1, LARP1, and METTL1 (**Figures 6A, B**). The risk score for OS in patients with HNSCC was determined as follows: (0.3372) * EIF3D + (0.2681)* EIF1 + (0.1292) * LARP1 + (0.0675) * METTL1. According to the risk score, HNSCC patients were divided into two groups. The distribution of risk scores, survival status, and expression levels of the four m7GRGs are shown in **Figures 6C, D**. Increased risk scores were associated with higher mortality risk and shorter survival time (**Figure 6C**). Kaplan-Meier curves showed lower OS rates in HNSCC patients with high risk scores [median time = 3 and 5.5 years, log-rank p = 0.000597, HR = 1.611 (1.227–2.115)] (**Figure 6D**). The 1-year, 3-year, and 5-year ROC curves had AUCs of 0.613, 0.613, and 0.612, respectively (**Figure 6E**).

### Internal and external validation of prognostic model

The prognostic model’s predictive value was validated using TCGA internal validation sets 1 and 2, showing the distribution of risk scores, survival time, and m7GRGs expression for each HNSCC patient (**Supplementary Figures S4A, B**). OS was significantly poorer in high-risk patients in the validation sets (**Supplementary Figures S4C**, **S3D**). AUCs for 1-year, 3-year, and 5-year OS were 0.621, 0.655, and 0.662 in TCGA validation set 1 (**Supplementary Figure S4E**) and 0.603, 0.613, and 0.660 in TCGA validation set 2 (**Supplementary Figure S4F**). The GSE65858, GSE41613, GSE85446 dataset was used as an external validation cohort, showing consistent results with the TCGA internal validation cohorts. Distribution of risk scores, survival time, and m7GRGs expression for each HNSCC patient are shown (**Supplementary Figures S4G–I**). OS was significantly lower in high-risk patients compared to low-risk patients (p < 0.05) (**Supplementary Figures S4J–L**). The AUCs for 1-year, 3-year, and 5-year OS were 0.627, 0.604, and 0.640 in the GSE65858 dataset, respectively (**Supplementary Figure S4M**). The AUCs for 1-year, 3-year, and 5-year OS were 0.673, 0.652, and 0.655 in the GSE41613 dataset, respectively (**Supplementary Figure S4N**). The AUCs for 1-year, 3-year, and 5-year OS were 0.660, 0.694, and 0.733 in the GSE85446 dataset, respectively (**Supplementary Figure S4O**). Collectively, these results validate the effectiveness of our risk score model, and the m7GRGs prognostic signature can predict OS in HNSCC.

### Construction of predictive nomogram

**Figure 7:**
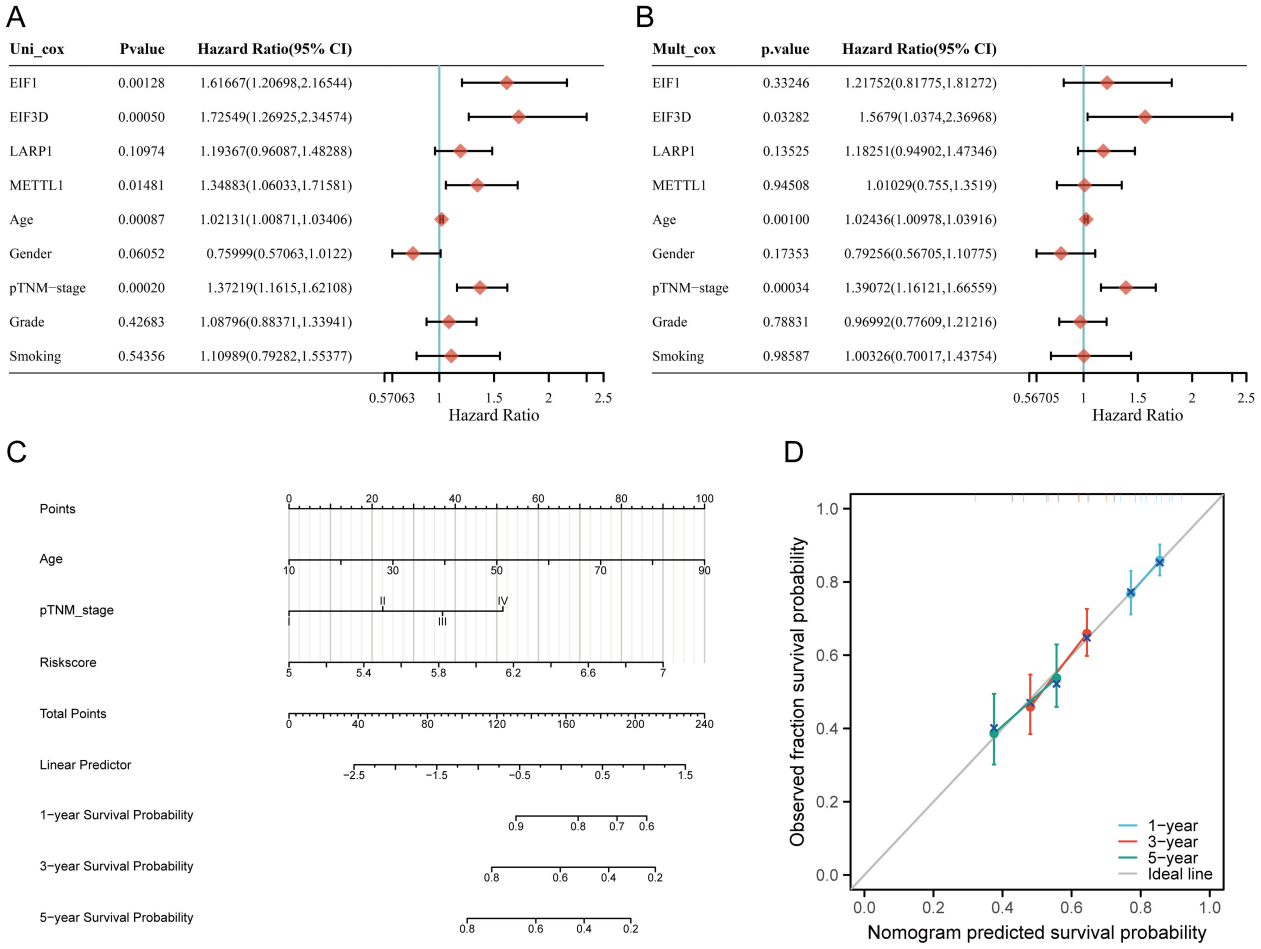
Construction of a predictive nomogram. **(A, B)** Hazard ratios and P-values for constituents involved in univariate and multivariate Cox regression analysis, considering clinical information and prognostic m7GRGs in HNSCC; **(C)** Nomogram predicting 1-, 3-, and 5-year OS of HNSCC patients; **(D)** Calibration curve of the OS nomogram model in the discovery group, with the diagonal dotted line representing the ideal nomogram.

Univariate and multivariate Cox analysis results indicate that age, tumor stage, and prognostic m7GRGs are independent prognostic factors for OS in HNSCC patients, based on multivariate Cox proportional hazards analysis (**Figures 7A, B**). Integrating risk scores with age and tumor stage-related prognostic independent factors, we established a nomogram to predict 1-year, 3-year, and 5-year OS.

Compared to other clinical features, the nomogram achieved the highest AUC values for predicting 1-year, 3-year, and 5-year overall survival (OS), with values of 0.788, 0.889, and 0.894, respectively (**Supplementary Figure S5A**). Additionally, a time-dependent AUC curve demonstrating the nomogram’s performance in predicting OS within the TCGA cohort was plotted (**Supplementary Figure S5B**). Decision curve analysis (DCA) was also conducted to assess the clinical utility of the nomogram (**Supplementary Figure S5C**). Compared to other clinical factors, the nomogram offered the best net benefit, with strong stability and reliability. These results suggest that the developed nomogram provides higher prognostic accuracy for HNSCC patients and may offer significant clinical benefits. The nomogram results showed that the prediction accuracy for 1-year, 3-year, and 5-year OS [C-index: 0.624 (0.601-0.646), p<0.001] (**Figures 7C, D**), PFS [C-index: 0.639 (0.585-1), p<0.001] (**Supplementary Figures S6A–D**), and DSS [C-index: 0.623 (0.571-1), p<0.001] (**Supplementary Figures S6E–H**) were more accurate than the ideal model.

### Correlation between m7GRG expression and HNSCC clinical pathological characteristics

In the TCGA cohort, there were notable differences in tumor grade and smoking status between the C1 and C2 subtypes. (**Supplementary Figure S7A**). Further analysis of TCGA data revealed that the expression of these four genes was significantly correlated with tumor grade (**Supplementary Figure S7B**) and varied notably across different pathological stages of HNSCC. Additionally, analysis using the UALCAN database confirmed that the expression levels of these four genes were significantly associated with HNSCC tumor stage and grade (**Supplementary Figure S7C**).

### Immune cell infiltration analysis

**Figure 8:**
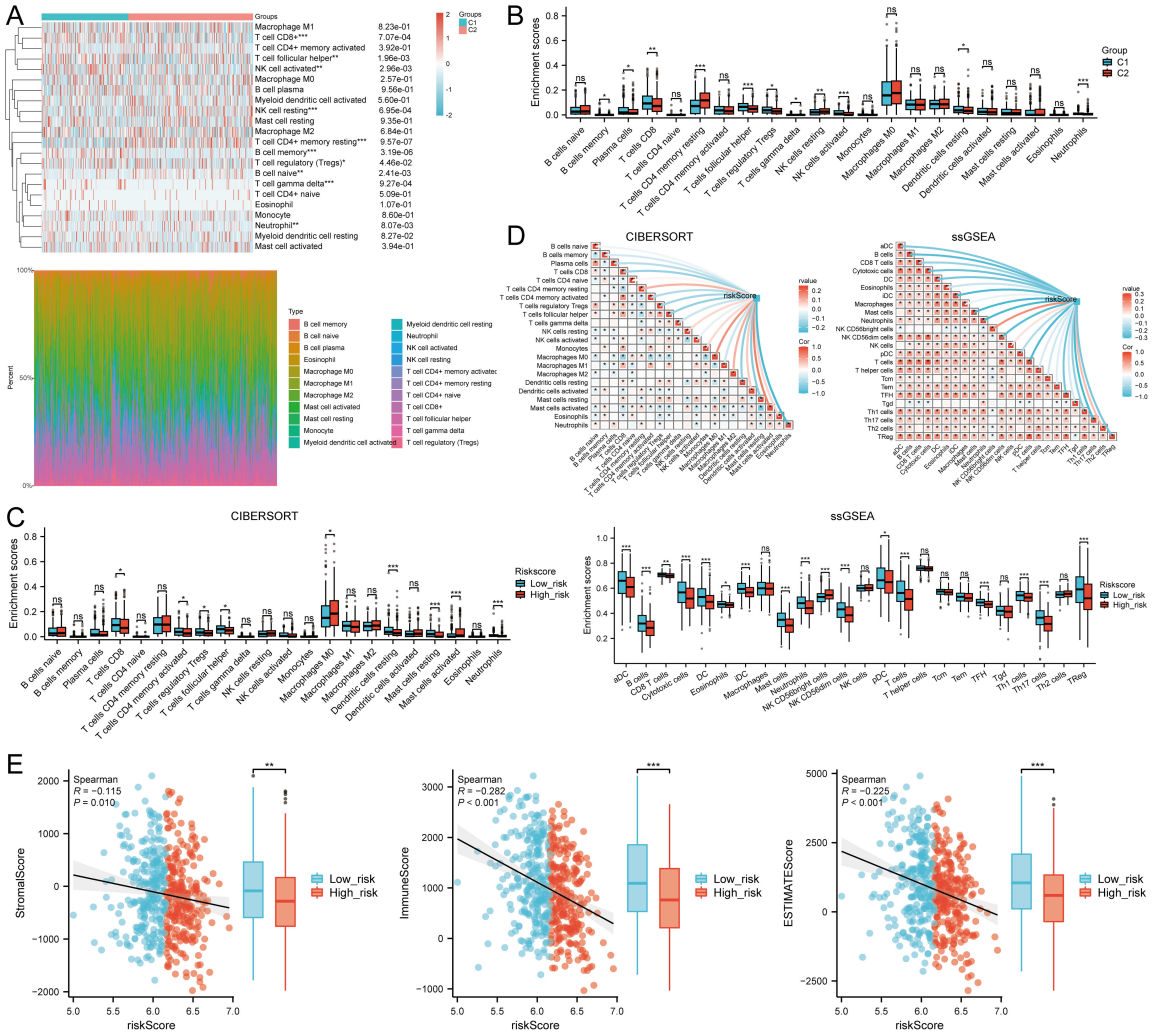
Relationship between m7GRG expression levels and immune infiltration in the tumor microenvironment. **(A)** Heatmap of immune cell scores between C1 and C2 subtypes in TCGA, along with the percentage abundance of tumor-infiltrating immune cells in each sample (CIBERSORT algorithm). **(B)** Differences in immune cell infiltration between Cluster 1 and Cluster 2 groups in HNSCC (CIBERSORT algorithm). **(C)** Differences in immune cell infiltration scores between high-risk and low-risk groups in HNSCC (CIBERSORT, ssGSEA algorithm). **(D)** Correlation between risk scores in HNSCC and tumor immune cell infiltration (CIBERSORT, ssGSEA algorithm). **(E)** Correlation between risk scores and three ESTIMATE metrics, and differences in ESTIMATE risk scores in HNSCC. n.s. no significance (p > 0.05), *p<0.05, **p<0.01, ***p<0.001.

The CIBERSORT algorithm revealed significant differences in the infiltration of various immune cell types, including CD8+ T cells, follicular helper T cells, activated NK cells, resting NK cells, resting memory CD4+ T cells, memory B cells, regulatory T cells (Tregs), naive B cells, gamma delta T cells, and neutrophils between the C1 and C2 HNSCC subtypes (**Figure 8A**). Additionally, the abundance of CD8+ T cells and activated NK cells in C1 was significantly higher than in C2, while the opposite trend was observed for resting memory CD4+ T cells (**Figure 8B**). Similar differences were observed using the quantTIseq, TIMER, EPIC, xCell, and MCPcounter algorithms (**Supplementary Figures S8A–E**). Quantitative analysis of immune cell infiltration using CIBERSORT and ssGSEA methods compared immune cell enrichment scores between high-risk (red) and low-risk (blue) groups. CIBERSORT showed that the low-risk group exhibited significantly higher infiltration of several immune cell types, including CD8+ T cells, activated CD4+ memory T cells, regulatory T cells (Tregs), follicular helper T cells, resting dendritic cells, resting mast cells, and neutrophils. Conversely, the high-risk group showed significantly higher enrichment levels of M0 macrophages and activated mast cells. These findings suggest that the high-risk group may exhibit a weaker antitumor immune response, yet show a stronger immune response in certain cell types, such as macrophages and activated mast cells (**Figure 8C**).

Analysis with CIBERSORT and ssGSEA revealed a significant correlation between immune cell infiltration and risk score (riskScore). Overall, risk scores were negatively correlated with the infiltration of several immune cell types, particularly plasma cells, CD8+ T cells, activated CD4+ memory T cells, regulatory T cells, follicular helper T cells, dendritic cells, mast cells, and neutrophils. This suggests that higher risk scores are associated with decreased infiltration of these immune cells, potentially linked to tumor immune evasion or increased immunosuppressive states. In contrast, risk scores showed a positive correlation with infiltration of certain immune cells, such as resting CD4+ memory T cells and macrophages, indicating enhanced infiltration of these cells in high-risk states. These findings provide new insights into the complex relationship between the tumor microenvironment and immune responses (**Figure 8D**).

Further analysis revealed a significant negative correlation between risk scores and stromal scores (P = 0.010, Cor = -0.115), immune scores(P <0.001, Cor = -0.282),and ESTIMATEScore (**Figure 8E**). TIMER analysis indicated that EIF1 negatively correlated with neutrophils, METTL1 with CD8+ T cells, and LARP1 positively correlated with multiple immune cell types, whereas EIF3D showed no significant correlation. However, their expression levels were closely associated with tumor purity (**Supplementary Figure S9A**). High levels of B cells, M2 macrophages, NK cells, CD8+ T cells, and Tregs were associated with improved prognosis, while elevated levels of M1 macrophages, neutrophils, and non-regulatory CD4+ T cells correlated with poorer overall survival (OS) rates (**Supplementary Figure S9B**). These findings suggest that m7GRGs are significantly linked to tumor immune infiltration, highlighting their potential as targets for immunotherapy.

### Immunotherapy response analysis

**Figure 9:**
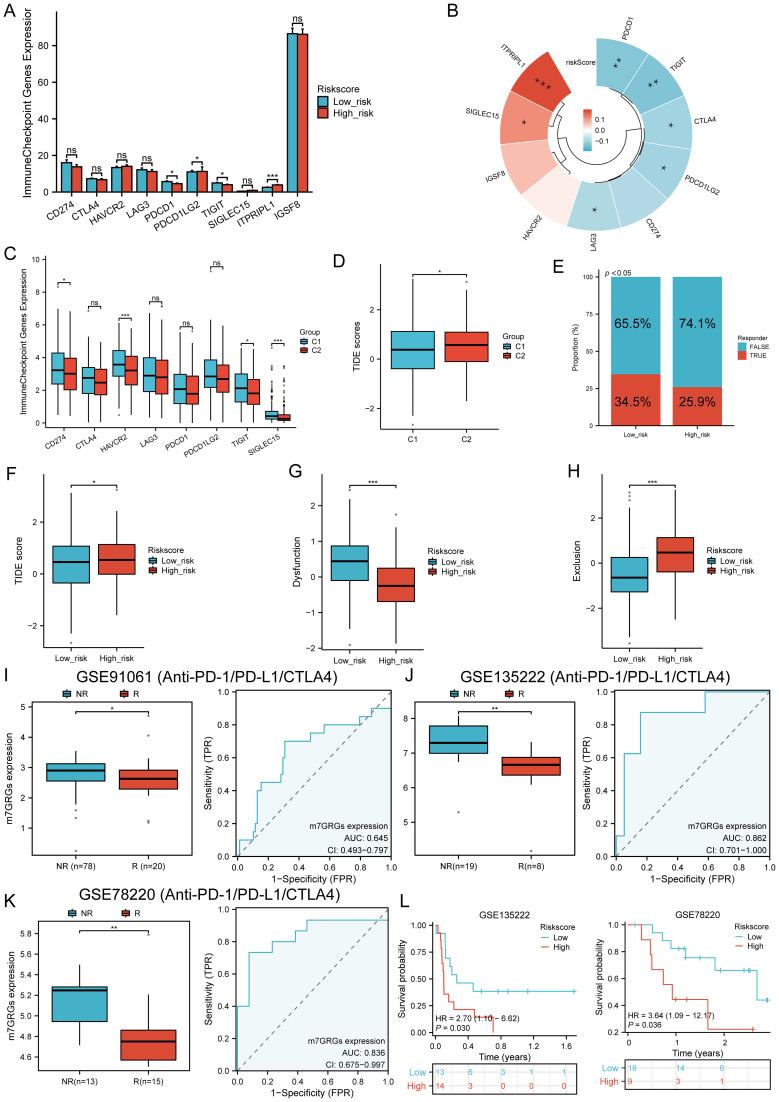
Immunotherapy response analysis. **(A)** Expression distributions of eight immune checkpoint-related genes between high and low m7GRG risk score groups in HNSCC; **(B)** Correlation between risk scores in HNSCC and immune checkpoint-related genes; **(C)** Expression distributions of eight immune checkpoint-related genes between cluster 1 and cluster 2 groups in HNSCC; **(D)** Differential reactions of cluster 1 and cluster 2 groups to immune checkpoint blocking in TIDE score; **(E)** Prediction of immunotherapy response rates in patients with high and low m7GRG risk scores; **(F)** Differential reactions of high and low m7GRG risk score groups to immune checkpoint blocking in TIDE score; **(G)** Differences in TIDE dysfunction score between high and low m7GRG risk score groups; **(H)** Differences in TIDE exclusion score between high and low m7GRG risk score groups; **(I-K)** Prediction of immune response and ROC analysis of m7GRG risk scores for predicting ICI responsiveness in GSE91061, GSE135222, and GSE78220 datasets; **(L)** Kaplan–Meier plots of overall survival for high and low risk patients in GSE135222 and GSE78220 datasets (NR, not responding to immunotherapy; R, responding to immunotherapy). n.s. no significance (p > 0.05), *p<0.05, **p<0.01, ***p<0.001.

The expression of eight immune checkpoint-related genes was assessed across two molecular subtypes and high- and low-risk groups. Significant expression differences were observed in PDCD1 (P < 0.05), PDCD1LG2 (P < 0.05), TIGIT (P < 0.05), and ITPRIPL1 (P < 0.001) between the high- and low-risk groups in HNSCC (**Figure 9A**). Risk scores were negatively correlated with CTLA4 (P = 0.0225, Cor = -0.102), LAG3 (P = 0.0458, Cor = -0.0891), PDCD1 (P = 0.0032, Cor = -0.1310), PDCD1LG2 (P = 0.0245, Cor = -0.1003), and TIGIT (P = 0.0021, Cor = -0.1369), but positively correlated with SIGLEC15 (P = 0.0101, Cor = 0.1146) and ITPRIPL1 (P < 0.00001, Cor = 0.1934) (**Figure 9B**). In the C1 subtype, the expression levels of CD274, HAVCR2, PDCD1, TIGIT, and SIGLEC15 were significantly higher than in the C2 subtype (**Figure 9C**). The TIDE score analysis revealed that the C1 subtype had significantly lower TIDE scores compared to the C2 subtype (**Figure 9D**). Additionally, predictions from the TIDE database indicated a higher proportion of immunotherapy responders in the low-risk group compared to the high-risk group (P < 0.05) (**Figure 9E**). The TIDE score was lower in the low-risk group (**Figure 9F**), with higher TIDE dysfunction scores (**Figure 9G**) and lower TIDE exclusion scores (**Figure 9H**). Validation using the GSE91061, GSE135222, and GSE78220 datasets confirmed the accuracy of m7GRGs expression in predicting immune response, with AUC values of 0.645 (95% CI, 0.493-0.797), 0.862 (95% CI, 0.701-1.000), and 0.836 (95% CI, 0.675-0.997), respectively (**Figures 9I–K**). In the GSE135222 and GSE78220 immunotherapy cohorts, OS was better in low-risk patients compared to high-risk patients (**Figure 9L**). These results suggest that patients with low m7GRGs risk scores are more likely to respond to immunotherapy and have better outcomes.

### TMB, MSI, mRNAsi, and drug sensitivity analysis

**Figure 10:**
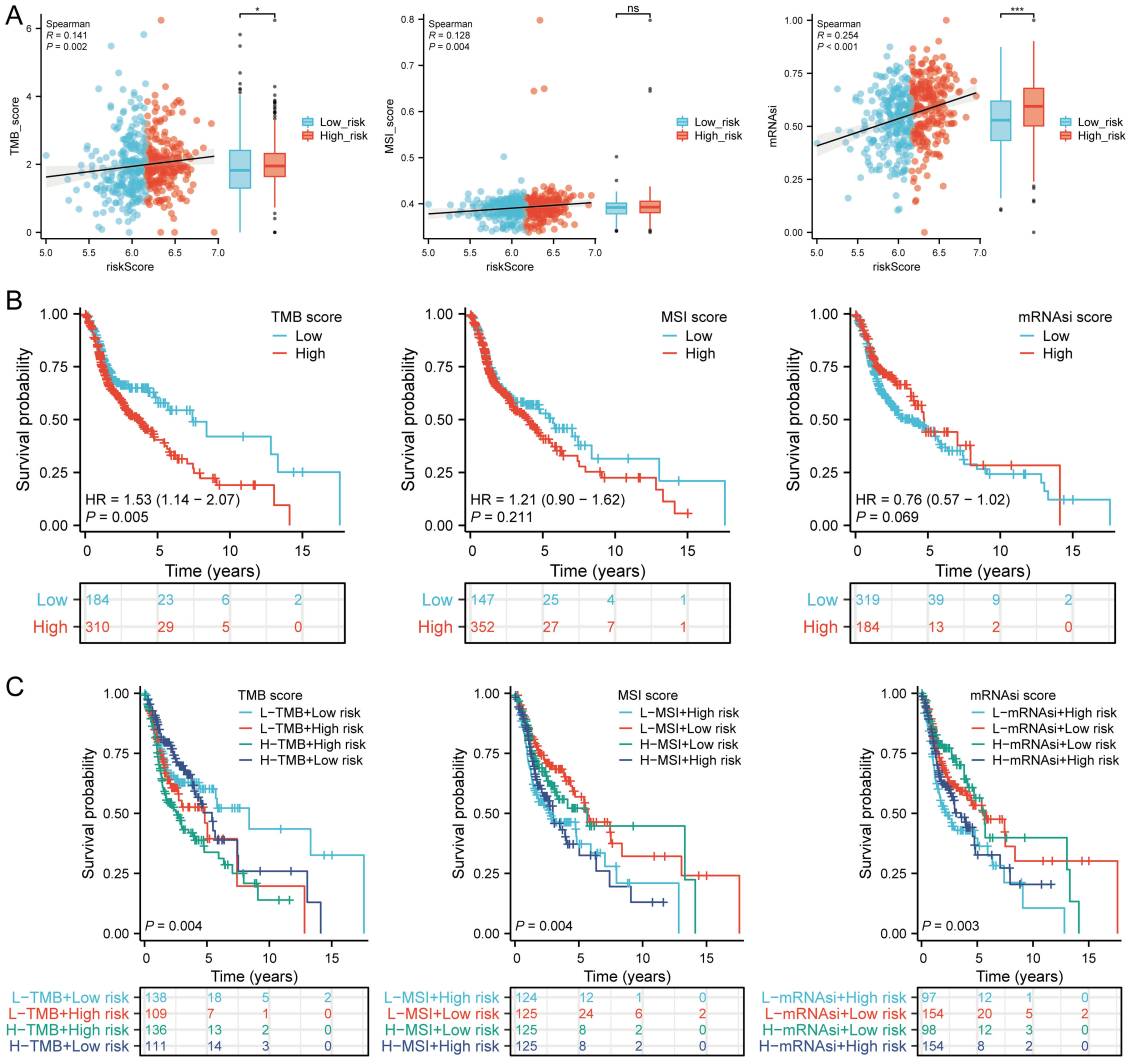
TMB, MSI, ESTIMATE, and mRNAsi. **(A)** Correlation between the risk score model and TMB, MSI, and mRNAsi, including differences in expression between high and low risk groups in HNSCC; **(B)** Kaplan-Meier curves for high and low TMB, MSI, and mRNAsi groups in HNSCC; **(C)** Kaplan-Meier curves for four groups classified by risk score, TMB, MSI, and mRNAsi in HNSCC. n.s. no significance (p > 0.05), *p<0.05, ***p<0.001.

**Figure 11:**
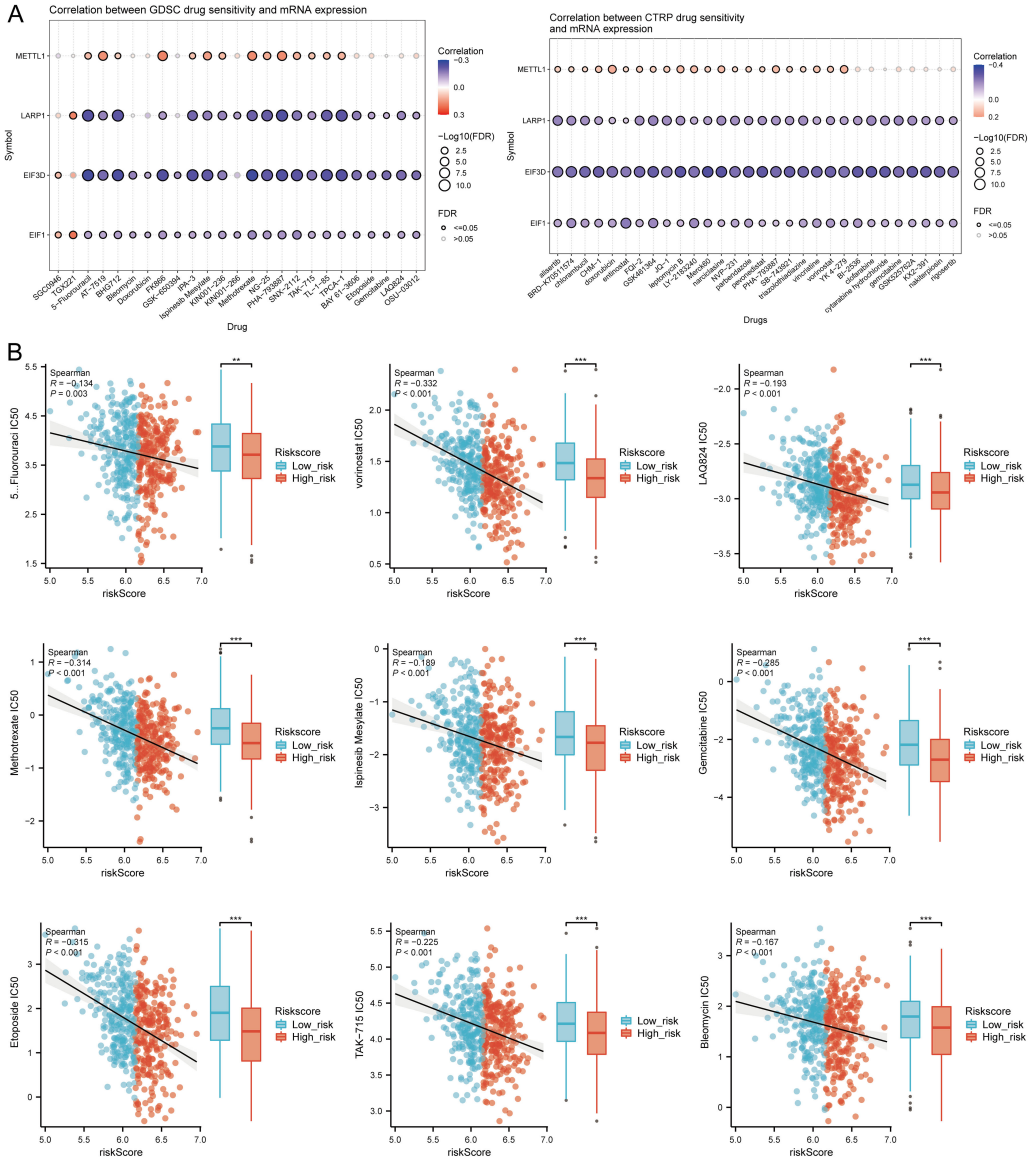
Drug sensitivity analysis. **(A)** Predictive antitumor drugs based on the risk score model in HNSCC from GDSC and CTRP datasets; **(B)** Spearman correlation analysis of IC50 scores and risk score model, and distribution of IC50 scores in high and low groups. **p<0.01, ***p<0.001.

TMB and mRNAsi scores were significantly higher in the high-risk group, with positive correlations between risk scores and TMB (R = 0.141, p = 0.002), MSI (R = 0.128, p = 0.004), and mRNAsi (R = 0.254, p < 0.001) (**Figure 10A**). Survival analysis showed that patients with high TMB scores had poorer OS (p = 0.005, HR = 1.53 [1.14 - 2.07]), but MSI scores (p = 0.211, HR = 1.21 [0.90 - 1.62]) and mRNAsi scores (p = 0.069, HR = 0.76 [0.57 - 1.02]) were not associated with prognosis (**Figure 10B**). Further analysis divided patients into four subgroups to assess the combined impact of risk scores and TMB on survival. OS was better in low TMB + low-risk score patients compared to high TMB + high-risk score patients (p = 0.004). Similarly, patients in the high MSI + high-risk group had poorer prognosis, while those in the low MSI + low-risk group had better OS (p = 0.004). Patients in the low mRNAsi + low-risk group had better OS compared to the high mRNAsi + high-risk group (p = 0.003) (**Figure 10C**). Several drugs from the GDSC and CTRP databases showed significant correlations with the risk score model (**Figure 11A**). High-risk HNSCC showed significantly higher sensitivity to 5-fluorouracil, vorinostat, LAQ824, methotrexate, ispinesib mesylate, gemcitabine, etoposide, TAK-715, and bleomycin. Spearman correlation analysis indicated negative correlations between risk scores and these drugs (**Figure 11B**). These drugs may be potential treatment options for HNSCC.

### Single-cell RNA data analysis

**Figure 12:**
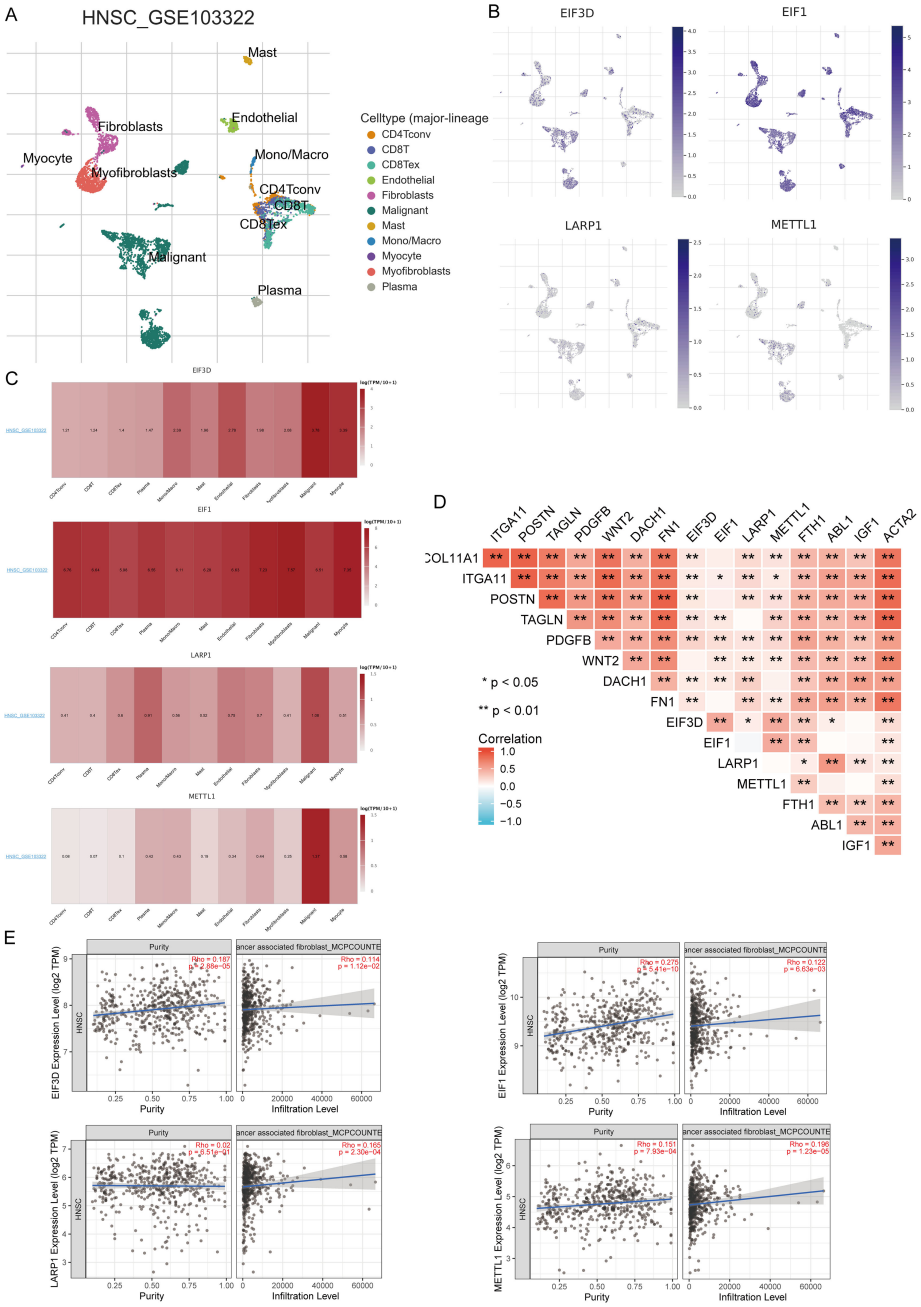
Expression of four prognostic m7GRGs in different immune cell types in HNSCC. **(A)** Cluster diagram of cell types in scRNA-seq data, with a t-SNE diagram showing expression of different immune cells (HNSCC_GSE103322) in HNSCC tissues; **(B)** Characteristic maps of four prognostic m7GRGs obtained from scRNA-seq data; **(C)** Heat maps of four prognostic m7GRGs from scRNA-seq data; **(D)** Correlation between expression of four prognostic m7GRGs and CAF-related markers; **(E)** Correlation between expression of four prognostic m7GRGs and CAF infiltration as analyzed by TIMER2.0. *p<0.05, **p<0.01.

In the TISCH database, we conducted single-cell RNA sequencing and clustering analysis using the GSE dataset (HNSCC_GSE103322) (**Figure 12A**). We evaluated the expression of EIF3D, EIF1, LARP1, and METTL1 at the single-cell level (**Figure 12B**) and observed strong expression in fibroblasts (**Figure 12C**). Further analysis revealed strong correlations between the expression of EIF3D, EIF1, LARP1, and METTL1 and CAF-related biomarkers (**Figure 12D**). Immune infiltration analysis showed significant correlations between CAF infiltration and the expression of EIF3D, EIF1, LARP1, and METTL1 (**Figure 12E**).

### Correlation analysis between m7GRGs and CRGs

We analyzed the correlation between prognostic m7GRGs and cuproptosis-related genes (CRGs) in the TCGA-HNSCC cohort. The results showed that prognostic m7GRGs were positively correlated with most CRGs. A heatmap was plotted to illustrate the correlation between m7GRGs and EIF3D, EIF1, LARP1, and METTL1 (**Supplementary Figure S10A**). Additionally, CRG expression levels were significantly higher in high-expression groups of EIF3D, EIF1, LARP1, and METTL1 (p < 0.05) (**Supplementary Figure S10B**). Further analysis identified DLD, PDHA1, PDHB, and GLS as key differentially expressed genes associated with prognostic m7GRGs (**Supplementary Figure S10C**). Kaplan-Meier curves indicated that high expression levels of PDHA1 and GLS were significantly linked to poorer prognosis in HNSCC (**Supplementary Figure S10D**). These findings suggest that prognostic m7GRGs are crucial in cuproptosis in HNSCC, potentially influencing HNSCC progression by regulating PDHA1 and GLS.

### Prediction and validation of upstream key miRNAs

Intersecting results from the ENCORI and RNAInter databases identified 1 EIF3D-miRNA, 66 EIF1-miRNAs, 127 LARP1-miRNAs, and 9 METTL1-miRNAs (**Supplementary Figure S11A**). A potential interaction network was constructed using Cytoscape software (**Supplementary Figure S11B**). Screening these candidate miRNAs for expression correlation in HNSCC via the Pan-cancer subproject of the ENCORI database revealed significant negative correlations in 13 EIF1-miRNAs, 21 LARP1-miRNAs, and 1 METTL1-miRNA interactions (**Supplementary Figure S12**). Further validation of the prognostic effects and expression levels of these miRNAs in HNSCC, using the Kaplan-Meier plotter and TCGA databases, showed that low expression levels of 10 miRNAs were significantly associated with poorer prognosis (**Supplementary Figure S13**). Notably, hsa-miR-30b-5p expression levels were significantly lower in HNSCC tissues compared to normal tissues (**Supplementary Figure S11C**). Furthermore, analysis using the Targetscan database revealed that the 3’-UTR of LARP1 contains a binding site for hsa-miR-30b-5p (**Supplementary Figure S11D**). Based on correlation, survival rate, and expression differential analysis, hsa-miR-30b-5p emerges as a potential miRNA in HNSCC.

### Prediction and validation of key miRNAs and potential lncRNAs

**Figure 13:**
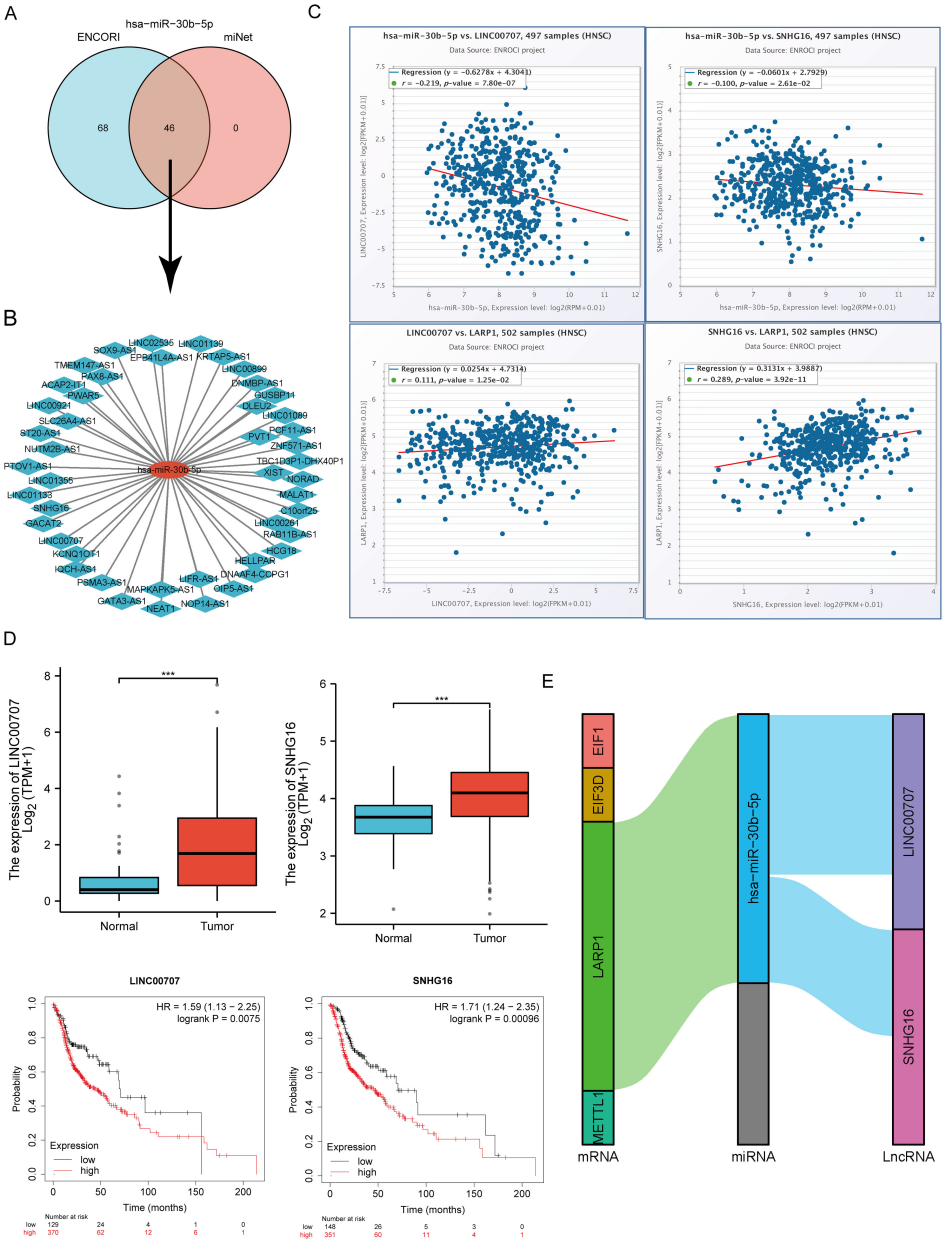
Screening of the LncRNA-miRNA-m7GRG regulatory axis in HNSCC. **(A)** Prediction of potential lncRNAs for hsa-miR-30b-5p through miRNet and ENCORI databases; **(B)** Construction of a potential miRNA-lncRNA network using Cytoscape software; **(C)** Correlation of two potential lncRNAs with hsa-miR-30b-5p and LARP1 in HNSCC; **(D)** Expression level and prognostic value of two potential lncRNAs in HNSCC; **(E)** Triple regulatory network of mRNA-miRNA-lncRNA affecting HNSCC prognosis. ***p<0.001.

Intersecting results from the ENCORI and miRNet databases predicted 46 lncRNAs binding to hsa-miR-30b-5p (**Figure 13A**). A miRNA-lncRNA regulatory network was established using Cytoscape software (**Figure 13B**). Correlation analysis of lncRNAs and hsa-miR-30b-5p expression using the ENCORI database identified significant correlations between LINC00707 and SNHG16 with hsa-miR-30b-5p and LARP1 (**Supplementary Table S4**, **Figure 13C**). Subsequent assessment of the prognostic value of these lncRNAs in HNSCC using the Kaplan-Meier plotter showed that LINC00707 and SNHG16 were significantly upregulated in HNSCC, and their upregulation was associated with poorer prognosis (**Figure 13D**). A key mRNA-miRNA-lncRNA regulatory network related to HNSCC prognosis was ultimately established, including four mRNAs (EIF3D, EIF1, LARP1, and METTL1), one miRNA (hsa-miR-30b-5p), and two lncRNAs (LINC00707 and SNHG16) (**Figure 13E**).

### Validation of m7GRGs at mRNA and protein levels

**Figure 14:**
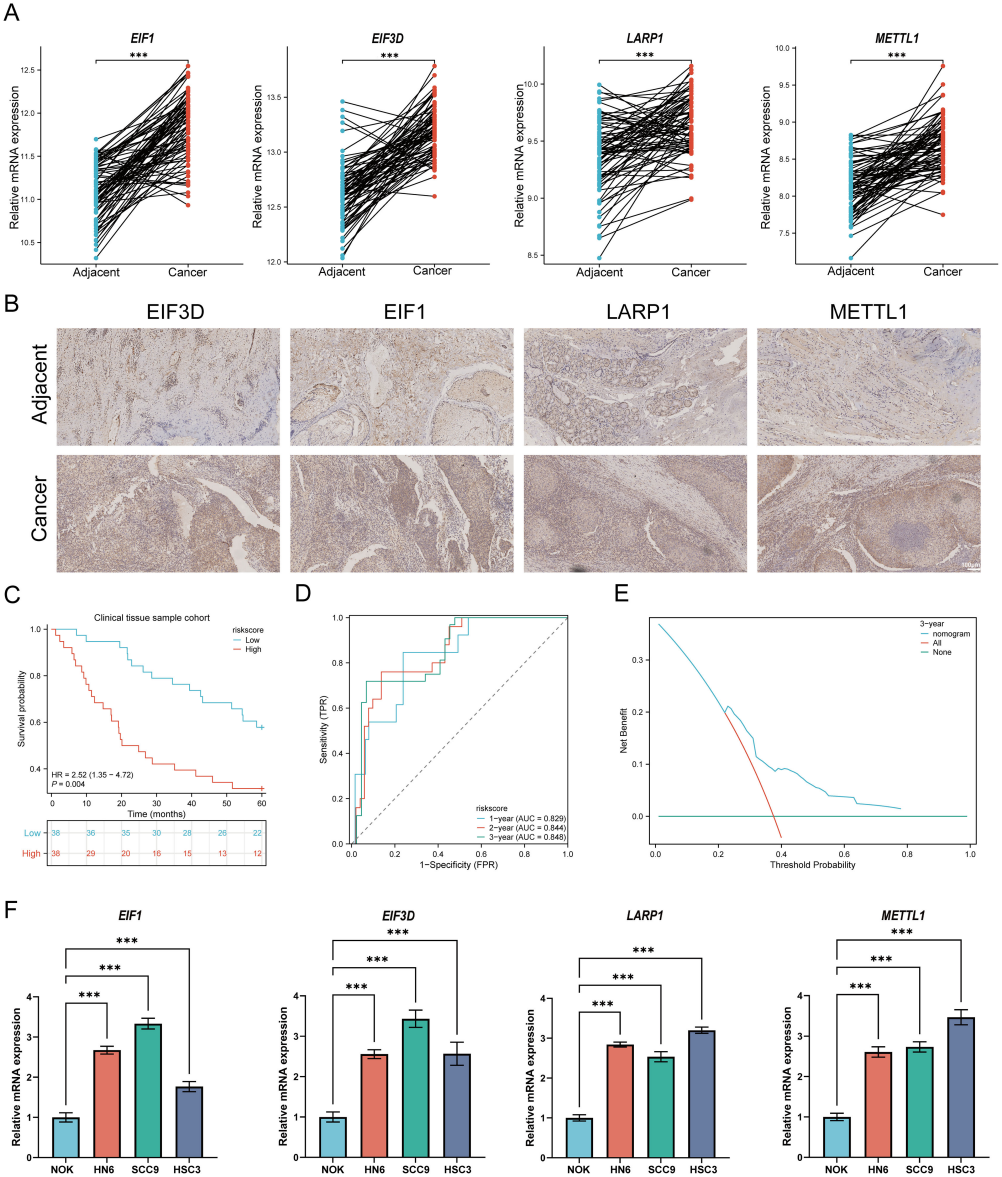
Cellular experiments and clinical sample validation. **(A)** Relative expression of prognostic m7GRGs in normal and HNSCC tissues; **(B)** Immunohistochemistry results of prognostic m7GRGs in normal and HNSCC tissues; **(C)** Overall survival curve for high/low-risk groups of HNSCC patients; **(D)** Time-dependent ROC curve for 1-, 3-, and 5-year OS for m7GRGs; **(E)** DCA curves for m7G-related prognostic signature in the clinical sample group; **(F)** Differential expression of four prognostic m7GRGs in NOK, NH12, CAL27, and SCC25 cell lines. ***p<0.001.

To validate the TCGA analysis results at the mRNA level, RT-qPCR was performed to assess the expression of four prognostic m7GRGs in HNSCC tissues and adjacent non-tumor tissues. Consistent with cellular expression patterns, EIF3D, EIF1, LARP1, and METTL1 were significantly upregulated in HNSCC tissues (**Figure 14A**). Immunohistochemistry results further corroborated these findings (**Figure 14B**). Additionally, quantitative immunohistochemical analysis revealed that the expression of EIF3D, EIF1, LARP1, and METTL1 in the tumor group was significantly higher than in adjacent tissues (**Supplementary Figure S14**). Additionally, the predictive performance of this prognostic model was validated using clinical tissue samples from our hospital. Patients were classified into high-risk and low-risk groups based on the risk scores derived from the established formula. Survival analysis revealed that high-risk patients had significantly shorter overall survival compared to the low-risk group (p = 0.004, HR = 2.52 [1.35–4.72], **Figure 14C**), consistent with the results from the TCGA and GEO databases. The AUCs for the 1-year, 3-year, and 5-year ROC curves were 0.829, 0.844, and 0.848, respectively (**Figure 14D**). Decision curve analysis (DCA) also demonstrated significant clinical utility in predicting survival rates (**Figure 14E**). Furthermore, RT-qPCR analysis of HNSCC cell lines revealed a significant upregulation of EIF3D, EIF1, LARP1, and METTL1 mRNA expression in HNSCC cell lines (NH6, HSC3, and SCC9) compared to normal epithelial cells (**Figure 14F**). The consistency between clinical tissues and cell line experiments confirmed the predictive reliability and validity of the constructed prognostic model for HNSCC patient prognosis.

### *In vitro* cell experiment of LARP1 in HNSCC

**Figure 15:**
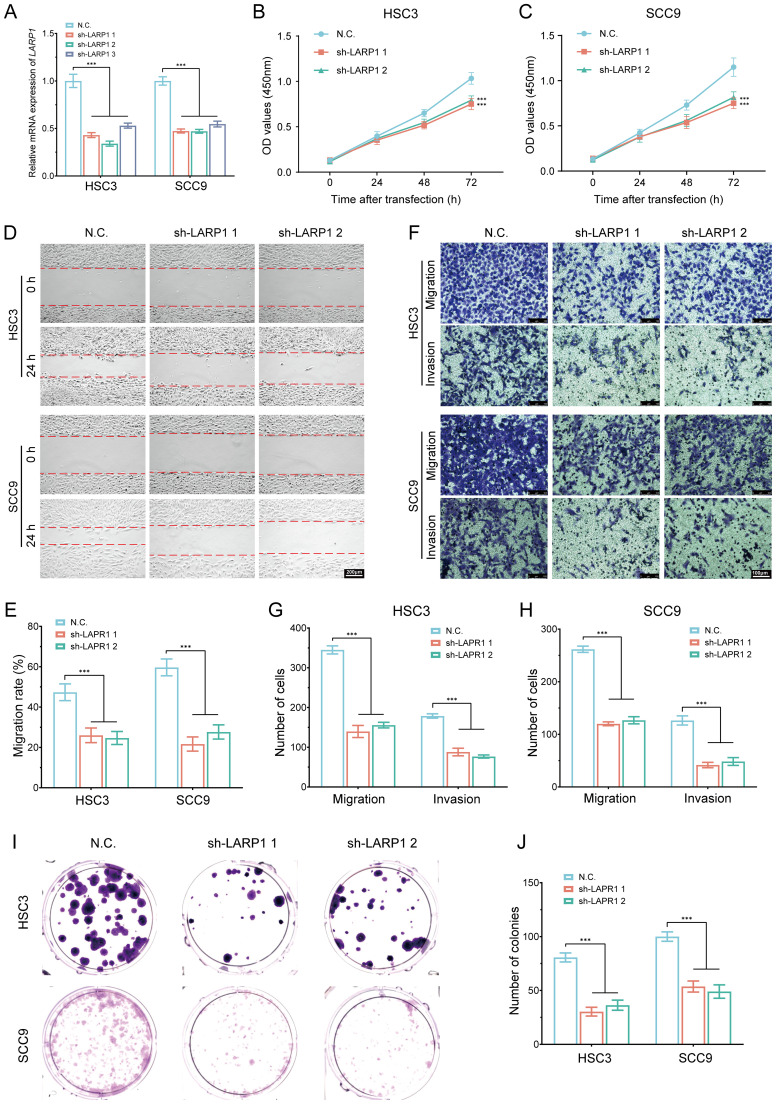
*In vitro* cell experiment of LARP1 in HNSCC. **(A)** RT-qPCR analysis showing the knockout efficiency of LARP1 in HSC3 and SCC9 cells; **(B, C)** CCK-8 assays performed in stable HSC3 and SCC9 cells with LARP1 knockdown; **(D, E)** Wound-healing assays in stable HSC3 and SCC9 cells with LARP1 knockdown; **(F-H)** Transwell migration and invasion assays in stable HSC3 and SCC9 cells with LARP1 knockdown; **(I, J)** Clone formation experiment following LARP1 knockdown in HSC3 and SCC9 cells. ***p<0.001.

To elucidate the role and functional significance of LARP1 in HNSCC, we performed LARP1 gene knockout experiments in HSC3 and SCC9 cells (**Figure 15A**). The CCK-8 assays revealed a significant reduction in the proliferation rates of both HSC3 and SCC9 cells following LARP1 knockout (**Figures 15B, C**). Additionally, wound healing and migration invasion assays demonstrated a marked decrease in the migration (**Figures 15D, E**) and invasion abilities (**Figures 15F–H**) of these cells. Colony formation assays further confirmed that LARP1 knockout significantly inhibited the proliferation of HSC3 and SCC9 cells (**Figures 15I, J**). Collectively, these findings suggest that LARP1 is crucial for the proliferation and metastatic potential of HNSCC cells.

## Discussion

This study systematically analyzes the expression and prognostic significance of m7GRGs in HNSCC. Using public database analysis, functional enrichment analysis, RT-qPCR validation, and LASSO-Cox regression analysis, we constructed a prognostic model based on m7GRGs. We evaluated its potential value in predicting HNSCC patient prognosis and immunotherapy response.

Firstly, we discovered widespread expression of m7GRGs in HNSCC, with most genes significantly upregulated in cancer tissues. The abnormal expression of these genes may be closely related to cancer development. Functional enrichment analysis revealed that m7GRGs are involved in several key biological pathways. These pathways play crucial roles in cancer development and progression, indicating that m7GRGs may promote HNSCC development by regulating these pathways. Current studies have confirmed that PPI is associated with the progression of HNSCC. PPI may play a role in the proliferation and migration of tumor cells. For example, EGFR (epidermal growth factor receptor) is a key protein commonly implicated in HNSCC (51). EGFR interactions are typically associated with downstream signaling pathways, such as PI3K/AKT (52) and MAPK (53), which regulate critical cellular processes like proliferation, survival, and migration. Overactivation of EGFR can result in rapid tumor cell proliferation and migration, thereby promoting the progression of HNSCC (54). The characteristics of the tumor microenvironment significantly influence key molecular targets for cancer therapy, and they are highly clinically relevant to treatment resistance and the response in HNSCC (55). Cyclins, such as Cyclin D1 and CDK4/6, and their interactions with other cell cycle-related proteins, may drive the accelerated proliferation of HNSCC cells (56). These interactions, identified through the STRING network, provide valuable insights into the dysregulation of cell cycle control and may contribute to understanding the mechanisms underlying tumor growth and metastasis. The PPI predictions from STRING can also reveal previously underexplored potential therapeutic targets. These targets may inhibit HNSCC progression by blocking key protein interactions. For example, targeting specific enzymes or transcription factors could suppress tumor cell proliferation and metastasis. Within the PPI network, EIF4E, NCBP2, and EIF4E3 exhibit high connectivity among all differentially expressed genes (DEGs). It has been reported that NCBP2 and EIF4E3 regulate the expression of CCL4/CCL5, influencing the immune microenvironment of HNSCC (57). EIF4E is overexpressed in HNSCC and exhibits oncogenic properties, playing a critical role in the progression of solid tumors (58). Furthermore, EIF4E expression is significantly correlated with both recurrence and relapse-free survival in HNSCC patients, with the PI3K/AKT/mTOR signaling pathway being highly activated (59). Arora et al. found that depletion of NCBP2 reduced the proliferation, migration, and invasion of oral squamous cell carcinoma cells (60). Therefore, these targets could block tumor cell proliferation and migration by inhibiting key interactions.

In this study, we observed a relatively low mutation rate for the genes under investigation, particularly for missense mutations. However, even these mutations, despite their lower frequency, could still have significant effects on tumor initiation and progression through a variety of mechanisms. Missense mutations are one of the most common types of genomic alterations found in tumors, and they can lead to diverse functional outcomes, including gain-of-function, loss-of-function, or neutral effects. For example, activating mutations in the PIK3CA gene have been extensively reported to aberrantly activate the PI3K/AKT signaling pathway, which in turn drives tumor cell proliferation and survival (61). Recent studies, such as those conducted by Rasti et al. (62), have provided further insights into the frequency and functional implications of PIK3CA activating mutations, particularly in breast cancer. These findings underscore the pivotal role of PIK3CA mutations in various tumor signaling pathways. On the other hand, missense mutations can also disrupt normal protein function, leading to a loss of biological activity. A well-known example of this is the TP53 gene, where missense mutations frequently result in the loss of function of the p53 protein. This loss impairs critical cellular processes, such as DNA repair and apoptosis, thereby facilitating tumorigenesis and cancer progression (63). In some cases, however, missense mutations might not significantly impact protein function, especially when the mutation occurs in non-essential domains of the protein. Such mutations may be relatively neutral and accumulate over time during tumorigenesis without directly contributing to the cancer-driving mechanisms (64). The focus of this study is on four m7GRG genes (EIF3D, EIF1, LARP1, and METTL1), which primarily harbor missense mutations. However, the precise functional effects of these mutations are still not fully understood. In the case of EIF3D, previous research has shown that mutations in this gene play a crucial role in the progression of colorectal cancer. Specifically, silencing the expression of EIF3D leads to a significant reduction in cell proliferation and colony formation, along with an excessive accumulation of cells in the cell cycle arrest and apoptosis phases (65). Moreover, EIF1 is involved in the initiation of translation, a fundamental cellular process. Although the precise effects of mutations in EIF1 are not completely clarified, it is hypothesized that mutations in this gene could influence translation efficiency, potentially disrupting the dynamic balance of protein synthesis (66). Similarly, mutations in LARP1, especially at the LARP1-T449 phosphorylation site, have been shown to play an important role in liver cancer. These mutations enhance translation, cell growth, migration, and invasion, while also regulating the expression of oncogenic proteins, thereby contributing to tumor progression. In the case of METTL1, missense mutations have been linked to diseases such as primary dwarfism and brain malformations, suggesting that these mutations could affect tRNA m7G methylation and, as a result, translation efficiency (67). Additionally, mutations in EIF4G1, another translation initiation factor, may alter the translation process and disrupt the dynamic balance of protein synthesis, further complicating cellular function (68). Finally, mutations in APAF1, a gene crucial for the apoptosis pathway, could lead to defects in the apoptotic cascade, disrupting the regulation of cell death, and promoting tumorigenesis and tumor progression (69). Although the exact functional effects of these mutations in the context of various cancers remain to be fully elucidated, future research should aim to investigate the specific roles of these mutations. This could be accomplished by using tools such as gene editing, cell-based models, and animal models to uncover how these mutations contribute to the initiation and progression of different types of cancer.

In recent years, several gene expression-based prognostic signatures have been developed and successfully applied in clinical trials and practice. These prognostic models play a pivotal role in quantifying patient risk, enabling the identification of high-risk patients and providing insights into their health status and potential treatment outcomes. Research has shown that prognostic signatures related to cuproptosis genes can effectively predict survival probabilities in hepatocellular carcinoma (HCC) patients (70). Similarly, disulfidptosis-related risk scores have been shown to accurately predict prognosis and response to immunotherapy in HNSCC (71). In the current study, we constructed a prognostic signature using LASSO-Cox regression. This model consisted of four genes: EIF3D, EIF1, LARP1, and METTL1. Kaplan-Meier survival analysis demonstrated that high-risk patients had a significantly worse prognosis compared to low-risk patients. The model’s robustness was further validated through ROC curves for 1-, 3-, and 5-year survival predictions. However, while some datasets showed AUC values above 0.8, other datasets exhibited AUC values below 0.8, revealing a variation in the classification performance across different datasets. To enhance the clinical utility and predictive accuracy of the model, we introduced a Nomogram. This Nomogram was constructed by integrating the risk score with additional clinical and pathological features, such as age and stage, based on the findings from multivariate Cox regression analysis. The results demonstrated that, compared to the risk score model, the Nomogram provided improved predictive accuracy, along with greater stability and reliability across various datasets.

The tumor microenvironment plays a crucial role in tumor progression and antitumor responses. In this study, we performed an in-depth analysis of immune cell infiltration in HNSCC using CIBERSORT and ssGSEA algorithms, revealing significant differences in immune cell infiltration patterns between distinct molecular subtypes (C1 and C2) as well as high- and low-risk groups. Our findings indicate that the C1 subtype and low-risk group exhibit stronger immune cell infiltration, particularly the active infiltration of CD8+ T cells and dendritic cells, which play key roles in tumor immune responses. CD8+ T cells directly kill tumor cells, while dendritic cells initiate adaptive immune responses by activating T cells (72, 73). These results corroborate previous studies, such as a recent study showing significantly elevated CD8+ T cell abundance in the low-risk group of HNSCC (74). This immune infiltration pattern is consistent with the characteristics of “hot tumors,” which typically exhibit robust immune cell activity and a higher immune response, enabling them to control tumor growth under immune surveillance. In contrast, patients in the high-risk group and the C2 subtype belong to the “cold tumor” category. Specifically, the tumor microenvironment in the high-risk group is more prone to developing an immunosuppressive state, hindering effective immune cell infiltration and allowing tumor cells to escape immune surveillance. In the high-risk group, the higher infiltration of M2 macrophages, which suppress CD8+ T cell function through the secretion of immunosuppressive factors, promotes tumor growth (75, 76). While some observations differ from pioneering results, such as the higher abundance of NK cells in the high-risk group, this may be attributed to hypoxic tumor microenvironments, which impair NK cell cytotoxic function (77). Thus, the high-risk status of m7GRGs may enhance immune evasion mechanisms, limiting immune cell functionality and diminishing the efficacy of immunotherapy.

In our study, we found a positive correlation between risk scores and NK cell infiltration, with higher NK cell levels associated with better prognosis, which aligns with previous research (78). However, the high levels of M2 macrophages and Tregs were associated with better prognosis. Existing studies suggest that M2 macrophages and Tregs are typically considered to promote immune suppression and are usually linked to poor prognosis (79, 80). This phenomenon may be attributed to the high heterogeneity of immune cell infiltration within the tumor microenvironment. The interactions between different immune cell types and their roles in different patients may vary. Under certain conditions, they may participate in milder immune regulatory responses, preventing excessive inflammation from damaging normal tissues and thus creating a favorable environment for antitumor immunity. For instance, Zhang J et al. (81) reported that neutrophils, activated mast cells, activated NK cells, resting memory CD4+ T cells, naive CD4+ T cells, M2 macrophages, and eosinophils are favorable factors for overall survival (OS) in HNSCC patients. Similarly, Zhu W.L. et al. (82) found that the expression of 24 immune cell subtypes (such as CD8+ T cells, dendritic cells, macrophages, NK cells, and activated NK T cells) was significantly higher in cluster 2 than in cluster 1, suggesting that cluster 2 may have a better immune therapy response and prognosis. Moreover, while high infiltration of M2 macrophages has been linked to immune tolerance and tumor immune escape, it could also be associated with better prognosis, particularly in tumor types with a more stable immune microenvironment (83). Therefore, these differences require further clinical validation to confirm our findings.

EIF3D, EIF1, LARP1, and METTL1 are highly expressed in various malignancies, participating in tumorigenesis through multiple mechanisms (84). For instance, EIF3D stabilizes GRK2 protein by blocking ubiquitin-mediated degradation, activating PI3K/Akt signaling, and promoting tumor cell proliferation and migration (85). EIF1 plays a crucial role in protein synthesis initiation, with gene mutations potentially linked to specific tumor risks (86). LARP1, an RNA-binding protein, is involved in various RNA metabolic processes within cells, such as RNA stability, processing, and translation (87). METTL1, an RNA methyltransferase, primarily catalyzes tRNA m7G modification, which is important for RNA stability, processing, and translation (67). Numerous studies have shown that METTL1 is significantly overexpressed in various malignancies and is associated with poor prognosis (88). Overall, the high expression of prognostic m7GRGs in malignancies, their association with poor prognosis, and their regulatory roles in tumorigenesis make them important targets for cancer diagnosis and treatment.

Post-transcriptional RNA modifications play crucial roles in shaping the tumor immune microenvironment (89). Studies have shown that m7GRGs are closely associated with the infiltration of immune cells such as CD8+ T cells, CD4+ T cells, NK cells, and macrophages. These immune cells participate in anti-tumor immune responses through various mechanisms, forming complex interactions with tumor cells and playing significant roles in tumor development and treatment. This association underscores the potential of m7GRGs in modulating the immune landscape of HNSCC, influencing treatment response and patient outcomes. In HNSCC patients, high stromal expression of the tumor-associated macrophage marker CD163+ predicts poor prognosis (90). Additionally, patients with high m7G-related risk exhibit decreased CD8+ T cell infiltration and increased Tregs and macrophage infiltration (91). Single-cell RNA sequencing data analysis indicates a significant association between poor prognosis m7GRGs and CAF infiltration. CAF, a major component of the tumor microenvironment, enhances immunotherapy efficacy by making tumor cells more recognizable and eliminable by the immune system. The discovery of new CAF subtypes may predict clinical responses to αPD-1 antibody in head and neck cancer patients (92). Therefore, combining CAF-targeted therapy with other treatments (such as chemotherapy, radiotherapy, and immunotherapy) can improve therapeutic outcomes.

ICIs have demonstrated significant clinical efficacy in various cancers, including advanced head and neck squamous cell carcinoma (HNSCC). ICIs activate T-cell-mediated antitumor responses by blocking tumor immune evasion mechanisms, such as PD-1/PD-L1 and CTLA-4 inhibitors. Although ICIs show promising prospects in HNSCC treatment, their efficacy is not universal, and therapeutic responses are influenced by several factors, including drug accessibility, economic costs, and patient adaptability to immunotherapy. Furthermore, the response rates to immunotherapy are not uniform, limiting the widespread application of ICIs. Therefore, the identification of novel biomarkers to improve treatment outcomes is crucial (93). This study proposes that m7GRGs are closely associated with the response to immunotherapy in HNSCC. Analysis of gene expression across different risk groups revealed significant differences in the expression of genes such as PDCD1, PDCD1LG2, TIGIT, and ITPRIPL1, indicating that low-risk patients may have a better response to immune checkpoint blockade (ICB) therapy. Using three independent immunotherapy datasets from the GEO database (targeting PD-1/PD-L1/CTLA-4), the study further validated that low-risk patients may serve as positive prognostic indicators for immunotherapy in HNSCC. These results suggest that m7GRGs could serve as potential biomarkers for predicting immunotherapy responses in HNSCC patients, offering new insights for personalized immunotherapy strategies. Although ICIs, such as pembrolizumab (Keytruda) and nivolumab (Opdivo), have shown validated efficacy in treating HNSCC, their application faces challenges in terms of economic cost and accessibility (94). However, we acknowledge that the correlation between immune checkpoint-related genes and risk scores was relatively weak in our study. Despite this, we believe that these preliminary observations provide valuable insights and suggest that the expression of certain immune checkpoint genes may exhibit some degree of variation between high-risk and low-risk groups. To further substantiate this hypothesis, we integrated subtype analysis, TIDE scores, and validation using independent datasets. These analyses confirmed that the proposed model still plays a significant role in immune microenvironment stratification and in predicting treatment response. This supports the notion that, while the immune checkpoint genes may not strongly correlate with the risk score in our study, they may still have relevant implications for the tumor’s immune landscape and therapeutic outcomes. Despite these promising findings, we are aware that the results might be influenced by factors such as small sample size or other confounding variables, which could limit the generalizability of our observations. To address this, future studies with larger sample sizes or additional experimental validations are essential to better understand the specific roles of immune checkpoint genes within the tumor immune microenvironment. Moving forward, we plan to conduct more extensive experiments with a larger cohort of patients to delve deeper into the relationships between immune checkpoints, tumor progression, and treatment response, and further validate our hypotheses. These future studies will help clarify the potential of integrating immune checkpoints into prognostic models and enhance our understanding of immune therapies in the context of tumor biology.

TMB and MSI are important biomarkers for assessing tumor immunotherapy responses and prognosis (95). High mRNAsi values are generally associated with higher malignancy, greater invasiveness, and worse prognosis, including shorter OS and PFS. Previous studies have indicated that Cancer Stem Cells (CSCs) are linked to tumor progression, drug resistance, and relapse (96). This study found that high expression of m7GRGs significantly increased the TMB, MSI, and mRNAsi scores in HNSCC. Therefore, a deeper analysis of the relationship between TMB, MSI, and mRNAsi with tumors can help in understanding the malignant mechanisms, assessing patient prognosis, identifying therapeutic targets, and predicting immunotherapy responses, thus supporting precision oncology. The study also revealed that the high-risk HNSCC group exhibited increased sensitivity to various chemotherapy and targeted drugs, although this relationship was weak (e.g., the correlation coefficients |r| for drugs like vorinostat, methotrexate, and gemcitabine ranged from 0.2 to 0.4). This suggests that drug sensitivity may be related to risk scores. Despite the modest correlation, the significance of the P-values indicates that this relationship is likely influenced by individual differences, drug mechanisms, and the immune microenvironment, necessitating further experimental validation. Furthermore, we found that m7GRGs were significantly correlated with genes related to cuproptosis. Studies have shown that changes in Pyruvate Dehydrogenase E1α subunit (PDHA1) trigger metabolic reprogramming and play a key role in the occurrence and development of HNSCC (97). Additionally, the upregulation of Glutaminase (GLS) is closely related to the clinical and pathological features of head and neck tumors (98). These findings suggest that the regulatory effects of m7GRGs on HNSCC may be associated with the copper-induced cell death mechanism, thereby influencing the progression and prognosis of HNSCC.

MicroRNAs have been shown to participate in various biological behaviors of different tumors through multiple signaling pathways. miR-30b-5p is a known microRNA that regulates gene expression by binding to the 3’ untranslated region (UTR) of target genes. It plays a crucial role in multiple biological processes, including cell proliferation, differentiation, migration, tumorigenesis, and immune regulation. miR-30b-5p inhibits the expression of the m7GRG gene by directly binding to the 3’ UTR of the LARP1 gene. It has been reported that miR-30b-5p is significantly downregulated in liver cancer tissues and cell lines, where it mediates DNMT3A inhibition of proliferation and targets USP37 to slow the cell cycle (99). Zhang et al. (100) reported that decreased miR-30b-5p expression may play a key role in cancer progression, particularly in tobacco-induced head and neck squamous cell carcinoma (HNSCC), and could serve as a novel biomarker and therapeutic target for this HNSCC subtype. miR-30b-5p acts as a tumor suppressor by targeting the G-protein subunit α-13 in renal cell carcinoma, influencing cell proliferation, metastasis, and epithelial-mesenchymal transition (101). It may also modulate the immune response within the tumor microenvironment by affecting the m7GRG gene. Additionally, studies indicate that miR-30b-5p targets USP22 to inhibit hypoxia-induced PD-L1 expression in lung adenocarcinoma cells (102). In this study, we discovered that the m7GRG gene is associated with tumor stemness, influencing self-renewal, drug resistance, and metastasis of tumor cells. Furthermore, miR-30b-5p may be involved in the regulation of cancer stem cell (CSC) characteristics. Cheng et al. (103) elucidated the role of miR-30b-5p in promoting lung cancer through the regulation of tumor stem cells.

Additionally, studies have reported that long non-coding RNAs (lncRNAs) play pivotal roles in cancer. In our study, LINC00707 and SNHG16 may act as molecular sponges, binding to hsa-miR-30b-5p, thereby relieving the inhibitory effect of hsa-miR-30b-5p on its target genes and upregulating the expression of m7GRGs. For example, LINC00707 promotes cervical cancer progression by regulating the miR-382-5p/VEGFA pathway (104) and interacts with Smad proteins to regulate TGFβ signaling and cancer cell invasion (105). LncRNA SNHG16 promotes colorectal cancer cell proliferation, migration, and epithelial-mesenchymal transition via miR-124-3p/MCP-1 (106). In hepatocellular carcinoma, HNF1A-AS1 acts as an oncogene and autophagy promoter by sponging has-miR-30b-5p, with the HNF1A-AS1-miR-30b axis being a key regulator of hepatocarcinogenesis (107). These findings suggest that these regulatory axes modulate the development of various cancers. We constructed a competing endogenous RNA (ceRNA) regulatory network and identified the lncRNA SNHG16/hsa-miR-30b-5p/LARP1 and lncRNA LINC00707/hsa-miR-30b-5p/LARP1 regulatory axes as being related to HNSCC patient prognosis.

In our preliminary bioinformatics analysis, LARP1 was found to be significantly associated with tumor progression, with higher expression levels observed in HNSCC. As an RNA-binding protein, LARP1 is involved in regulating various cellular processes and has been shown in multiple studies to play a crucial role in tumor cell migration, proliferation, and invasion, particularly during tumor metastasis. Therefore, its pivotal role in our computational model makes LARP1 a prime candidate for validation. Furthermore, survival analysis demonstrated a significant correlation, with high expression of LARP1 associated with poorer overall survival (HR = 1.422, p = 0.0105), further supporting the close relationship between LARP1 and tumor prognosis. Immune infiltration analysis and transcriptomic data also revealed significant associations between LARP1 and immune cell infiltration in the tumor microenvironment, as well as tumor progression, enhancing its reliability as a potential oncogene. Additionally, our cellular experimental results showed relatively high expression levels of LARP1 across various cell lines, and in clinical tissue samples, LARP1 expression was significantly higher than in normal tissues or the other three candidate genes. Among the predicted regulatory axes, pathways such as LINC00707/hsa-miR-30b-5p/LARP1 and SNHG16/hsa-miR-30b-5p/LARP1 showed stronger associations compared to other candidate genes, highlighting their multifaceted regulatory potential in tumors. Based on these findings, we selected LARP1 as the primary gene for validation. Gene knockout experiments further verified LARP1’s function in HNSCC cells, revealing that LARP1 knockout significantly inhibited HNSCC cell proliferation, migration, and invasion. Further research into the specific mechanisms of these networks could unveil new therapeutic targets and advance personalized treatment.

LARP1, an RNA-binding protein, is involved in regulating various cellular processes. It is known to modulate the stability and translation of mRNA, particularly those genes associated with the cell cycle. By binding to mRNA, LARP1 promotes the synthesis of certain cell cycle-related proteins, thereby influencing cell proliferation and division. Burrows et al. (108) demonstrated that LARP1 is part of complexes with PABP and eIF4E, playing an essential role in orderly mitosis, cell survival, and migration. Silencing LARP1 expression via siRNA reduces the overall protein synthesis rate, leading to mitotic arrest and delayed cell migration. These findings suggest that LARP1 contributes to the synthesis of proteins necessary for cell remodeling and migration. In our *in vitro* experiments, we found that LARP1 knockout significantly inhibited the migration and invasion of HNSCC cells. LARP1 may regulate the expression of genes related to cell adhesion, matrix degradation, and migration by interacting with specific transcription factors or signaling pathways. These processes are crucial for tumor metastasis. Hsa_circRNA_002144 promotes colorectal cancer growth and metastasis through the miR-615-5p/LARP1/mTOR pathway (109). Desi et al. (51) identified a positive feedback loop between LARP1 and MYC, which promotes tumorigenesis. LARP1 also regulates mitochondrial oxidative phosphorylation in response to the PI3K/mTOR pathway, contributing to ovarian cancer cell survival (110). The downregulation of KCNQ1OT1 inhibits proliferation, invasion, and drug resistance in osteosarcoma cells through miR-129-5p-mediated LARP1 regulation (111). Additionally, single-cell sequencing in the study revealed that high expression of LARP1 is closely associated with cancer-associated fibroblasts (CAFs), which are known to play a key role in tumor immune evasion and metastasis. We further explored LARP1’s role in the tumor immune microenvironment, including its potential impact on immune cell recruitment and immune suppression. Research indicates that LARP1 is associated with the infiltration of various immune cells and may facilitate the conversion of “cold” tumors to “hot” tumors in liver cancer (109). In future studies, we plan to investigate the synergistic effect of LARP1 with other known prognostic molecules (such as EIF3D, EIF1, METTL1) in HNSCC, particularly their potential interactions in tumor cell growth, metastasis, and immune evasion.

## Conclusion

In summary, this study demonstrates that high expression of m7GRGs (EIF3D, EIF1, LARP1, and METTL1) in HNSCC patients is significantly associated with clinicopathological features, prognosis, epigenetics, CRG expression, and TME. Moreover, our findings establish a theoretical framework for future HNSCC immunotherapy. Additionally, LARP1 is experimentally validated as a key promoter of HNSCC progression by enhancing tumor cell proliferation, migration, and invasion. Potential lncRNA SNHG16/hsa-miR-30b-5p/LARP1 and lncRNA LINC00707/hsa-miR-30b-5p/LARP1 regulatory networks may offer new therapeutic targets, aiding in the development of personalized HNSCC treatments.

## Data Availability

The original contributions presented in the study are included in the article/**Supplementary Material**. Further inquiries can be directed to the corresponding authors.
