# Supplementary material for: Integrative analysis of m7G methylation-associated genes prognostic signature with immunotherapy and identification of LARP1 as a key oncogene in head and neck squamous cell carcinoma
Source: Front Immunol. 2025 Feb 13;16:1520070. doi: 10.3389/fimmu.2025.1520070 (PMC11864954; doi:10.3389/fimmu.2025.1520070)
Supplement: Supplementary file 1 [file DataSheet1.pdf]

SUPPLEMENTAL DATA

Table S1. The clinical characters of HNSCC patients in TCGA cohort

Clinical characters	Number
Alive	286
Dead	218
Mean (SD)	60.95<br>(12)
FEMALE	134
MALE	370
AMERICAN INDIAN	2
ASIAN	10
BLACK	47
WHITE	430
T1	34
T2	146
T3	133
T4	25
T4a	152
T4b	3
TX	11

N0	242
N1	81
N2	19
N2a	18
N2b	77
N2c	41
N3	7
NX	19
M0	479
M1	5
MX	20
I	25
II	81
III	91
IVA	291
IVB	13
IVC	3
G1	62
G2	301
G3	119
G4	2

GX	17
Metastasis	19
Primary	9
Recurrence	39
Non-smoking	113
Smoking	381
Non-radiation	63
Radiation	121
Neoadjuvant	10
No neoadjuvant	494
Chemotherapy	159
Chemotherapy:Immunotherapy	3
Chemotherapy:Other. specify in notes	1
Chemotherapy:Targeted Molecular therapy	7
Chemotherapy:Targeted Molecular therapy:Vaccine	1
Immunotherapy	2

Table S2. Primer sequences of genes

Real-time quantitative PCR primer sequence	
**Gene**	Sequence (5'- 3' on minus strand)
GAPDH	Fwd: GGAGCGAGATCCCTCCAAAAT
	Rev: GGCTGTTGTCATACTTCTCATGG
EIF3D	Fwd: TGACACCCGTGATCCAGGA
	Rev: TGGTAGGGCATATCCCGAAAC
EIF1	Fwd: GAAACGGCAGGAAGACCCTTA
	Rev: CGGATGCTCAATTACAGTACCAT
LARP1	Fwd: AGGCTCCCATACCTAGCTGC
	Rev: TGCCTTTGCGCTGTTTAGGAA
METTL1	Fwd: GGCAACGTGCTCACTCCAA
	Rev: CACAGCCTATGTCTGCAAACT

Table S3. Gene sets enriched in phenotype high.

ID	NES	p.adjust	FDR
KEGG_DNA_REPLICATION	-2.152431	1.01e-05	0.0002
KEGG_CELL_CYCLE	-1.901104	1e-05	0.0002
KEGG_ECM_RECEPTOR_INTERACTION	-1.630998	1e-05	0.0002
KEGG_FOCAL_ADHESION	-1.519811	1e-05	0.0002
KEGG_PATHWAYS_IN_CANCER	-1.448553	1e-05	0.0002
KEGG_P53_SIGNALING_PATHWAY	-1.476550	0.0011	0.0067

KEGG_WNT_SIGNALING_PATHWAY	-1.354982	0.0014	0.0076
KEGG_NOTCH_SIGNALING_PATHWAY	-1.500272	0.0029	0.0135
KEGG_ERBB_SIGNALING_PATHWAY	-1.355811	0.0073	0.0275
KEGG_TGF_BETA_SIGNALING_PATHWAY	-1.354860	0.0080	0.0290

Table S4. Correlation analysis between lncRNA and hsa-miR-30b-5p or lncRNA and LARP1 in HNSCC determined by ENCORI and TCGA databases.

LncRNA	miRNA	R value	p value
NOP14-AS1	hsa-miR-30b-5p	-0.173	1.05E-04
LINC02535	hsa-miR-30b-5p	-0.201	6.18E-06
LINC00707	hsa-miR-30b-5p	-0.219	7.80E-07
SNHG16	hsa-miR-30b-5p	-0.100	2.61E-02
**LncRNA**	**mRNA**	**R value**	**p value**
NOP14-AS1	LARP1	-0.028	5.37E-01
LINC02535	LARP1	-0.029	5.14E-01
LINC00707	LARP1	0.111	1.25E-02
SNHG16	LARP1	0.289	3.92E-11

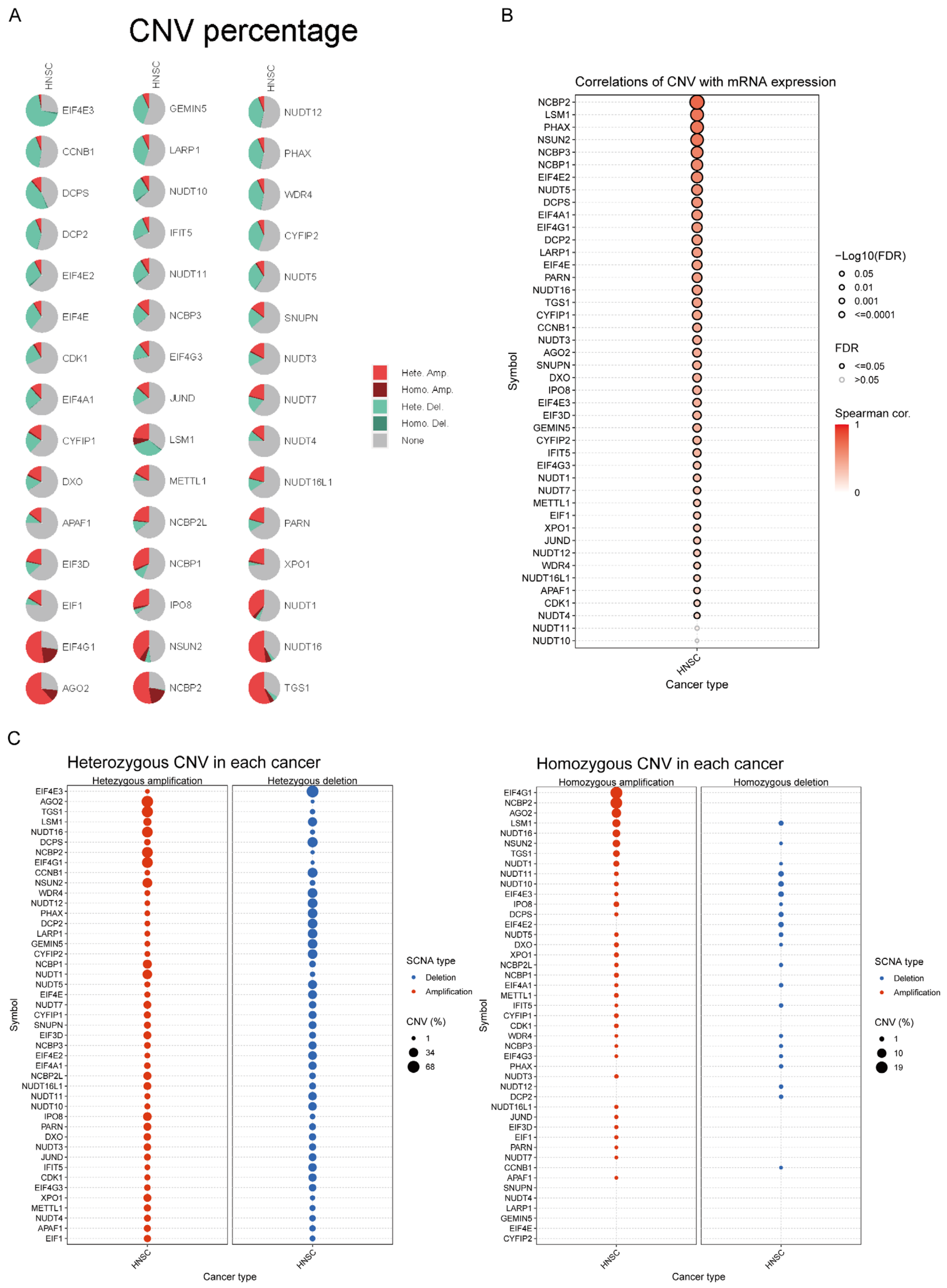

Figure S1. Analysis of copy number variation (CNV) of m7GRGs in HNSCC. (A) CNV pie chart distribution; (B) CNV expression levels of 45 m7GRGs in HNSCC cohort; (C) heterozygous CNV and homozygous CNV profiles of the 45 m7GRGs in HNSCC cohort.

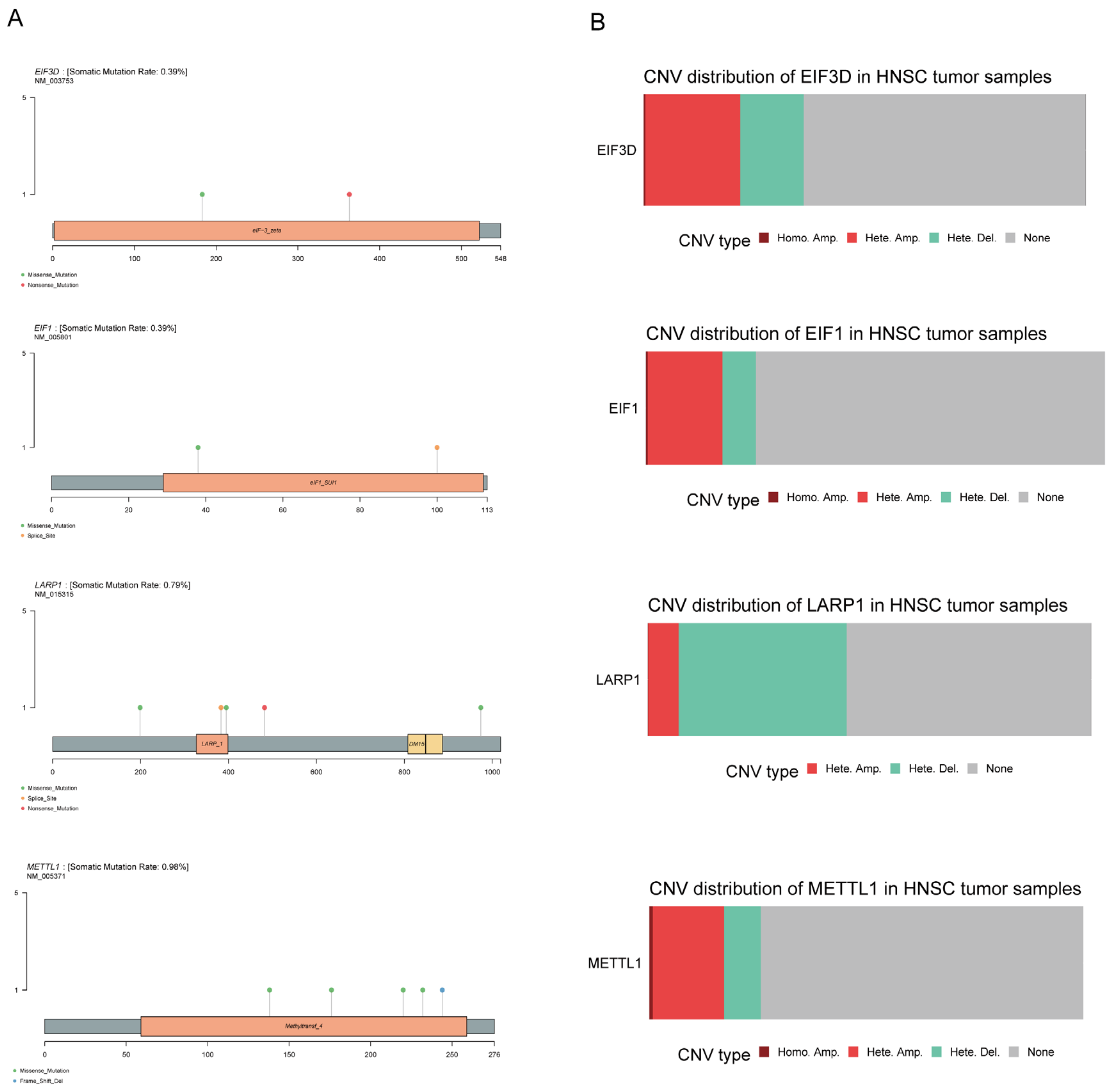

Figure S2. Distribution of m7GRG mutations associated with patient prognosis. (A) Mutation sites; (B) Detailed expression levels of m7GRGs in HNSCC tumor samples with CNV.

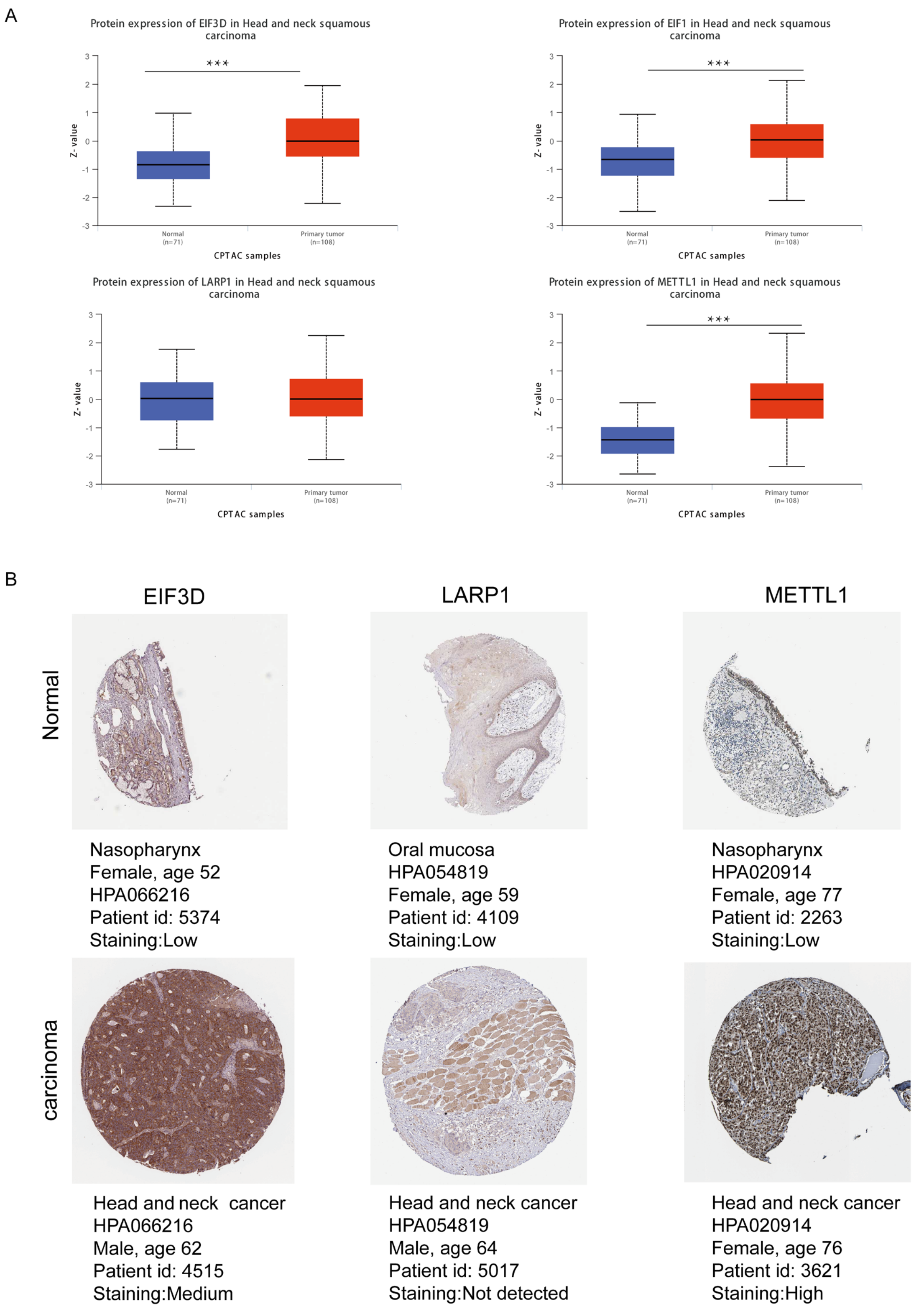

Figure S3. Differential protein expression of four prognostic m7GRGs in normal and HNSCC tissues. (A) Protein expression of prognostic m7GRGs in HNSCC tissues (UALCAN); (B) Immunohistochemical data of prognostic m7GRGs in HNSCC tissues obtained from HPA (magnification, 200×).

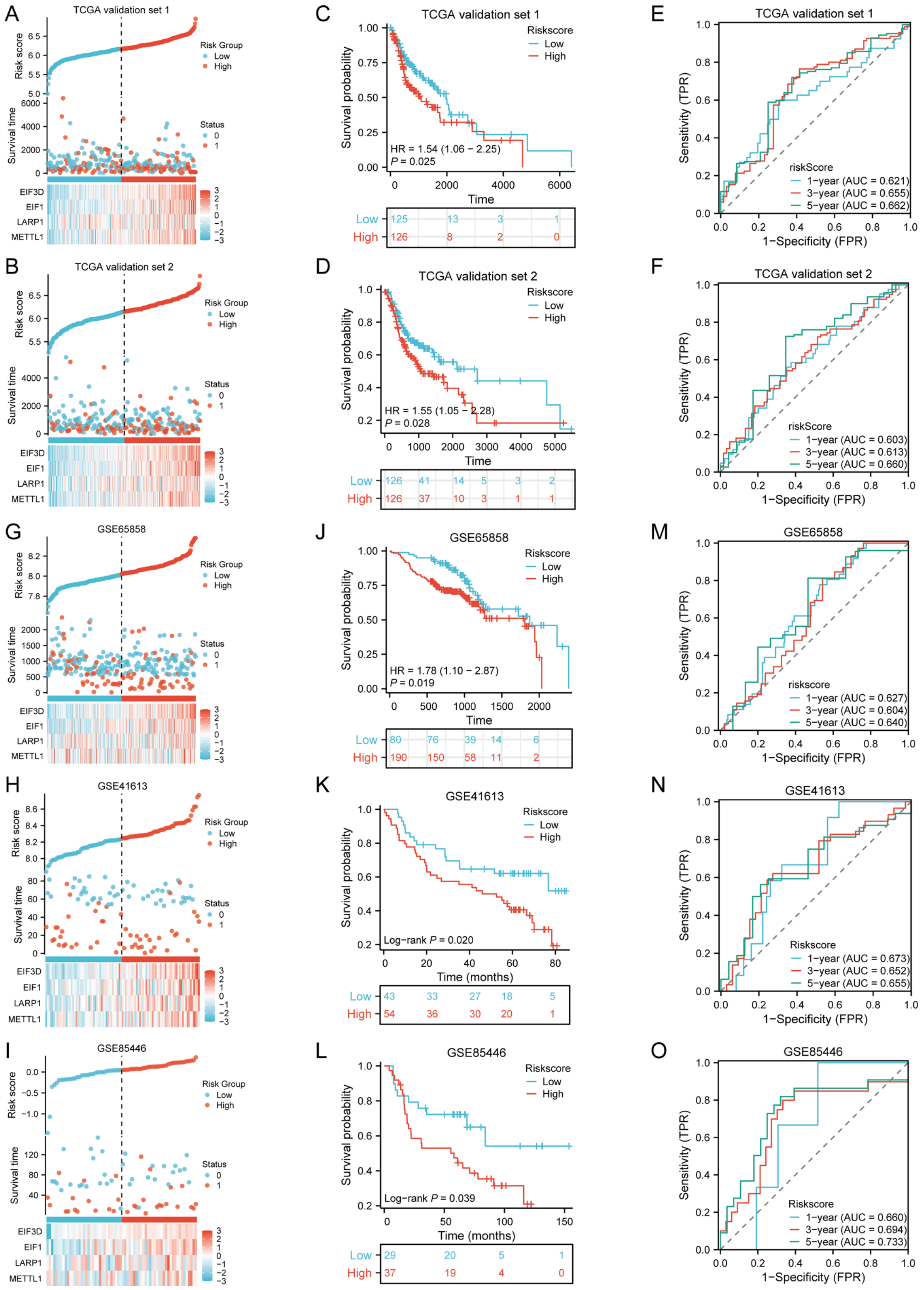

Figure S4. Prognostic value of DRGs signature and risk score validation in HNSCC patients. (A, B) Distribution of risk score, survival status, and expression of prognostic DRGs for patients in low- and high-risk groups in TCGA validation set 1 and TCGA validation set 2. (C, D) Risk score and survival probabilities in TCGA

validation set 1 and TCGA validation set 2. (E, F) Time-dependent ROC curve analyses of risk score in TCGA validation set 1 and TCGA validation set 2. (G-I) Distribution of risk score, survival status, and expression of prognostic DRGs in HNSCC in GSE65858, GSE41613, GSE85464 dataset; (J-L) OS curve of HNSCC patients in high/low-risk groups in GSE65858, GSE41613, GSE85464 dataset; (M-O) Time-dependent ROC curve for 1-, 3-, and 5-year OS for DRGs in GSE65858, GSE41613, GSE85464 dataset.

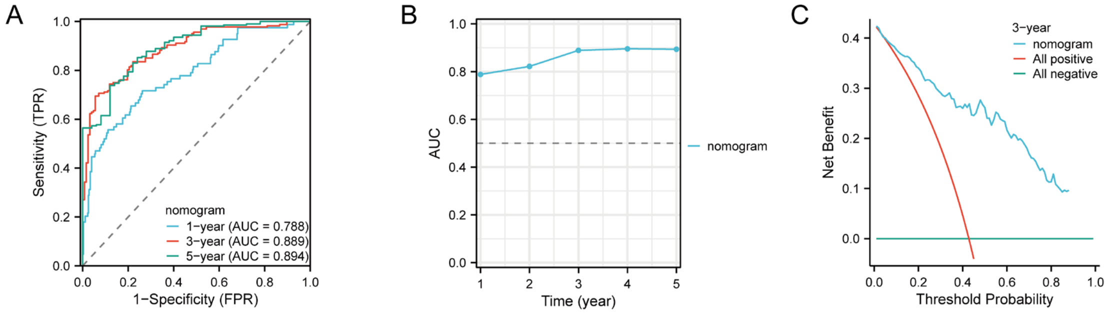

Figure S5. Nomogram prediction efficiency. (A) ROC curves for predicting 1-, 3-, and 5-year OS. (B) Time-dependent AUC curve shows the nomogram to predict OS performance. (C) DCA curves for the nomogram.

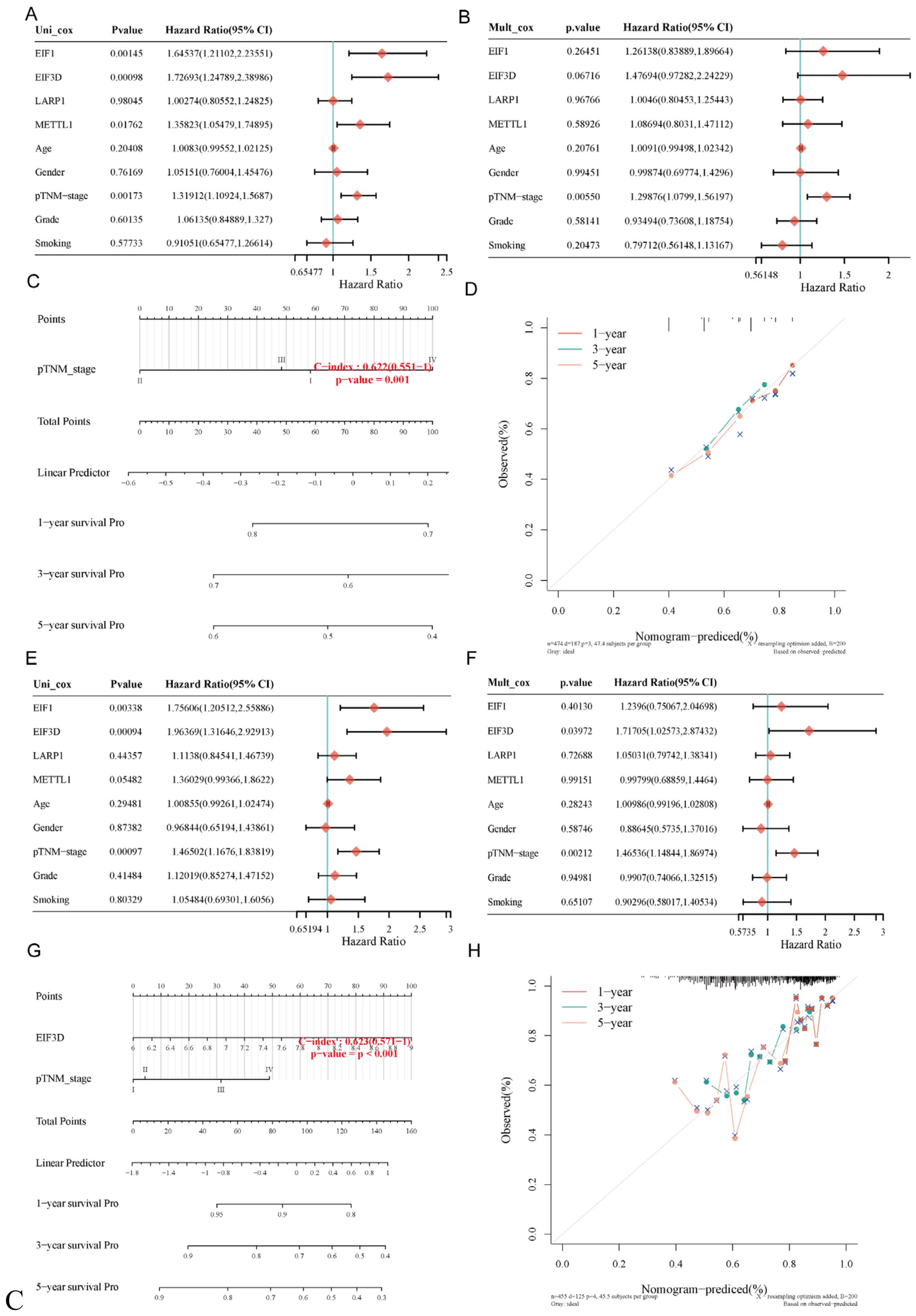

Figure S6. Construction of a predictive nomogram (PFS, DSS). (A, B)Univariate and multivariate Cox regression analysis of clinicopathological features and m7GRGs in HNSCC patients for PFS; (C, D) Nomogram for predicting 1-, 3-, and 5-year PFS of HNSCC patients; (E, F) Univariate and multivariate Cox regression analysis of

clinicopathological features and m7GRGs in HNSCC patients for DSS; (G, H) Nomogram for predicting 1-, 3-, and 5-year DSS of HNSCC patients.

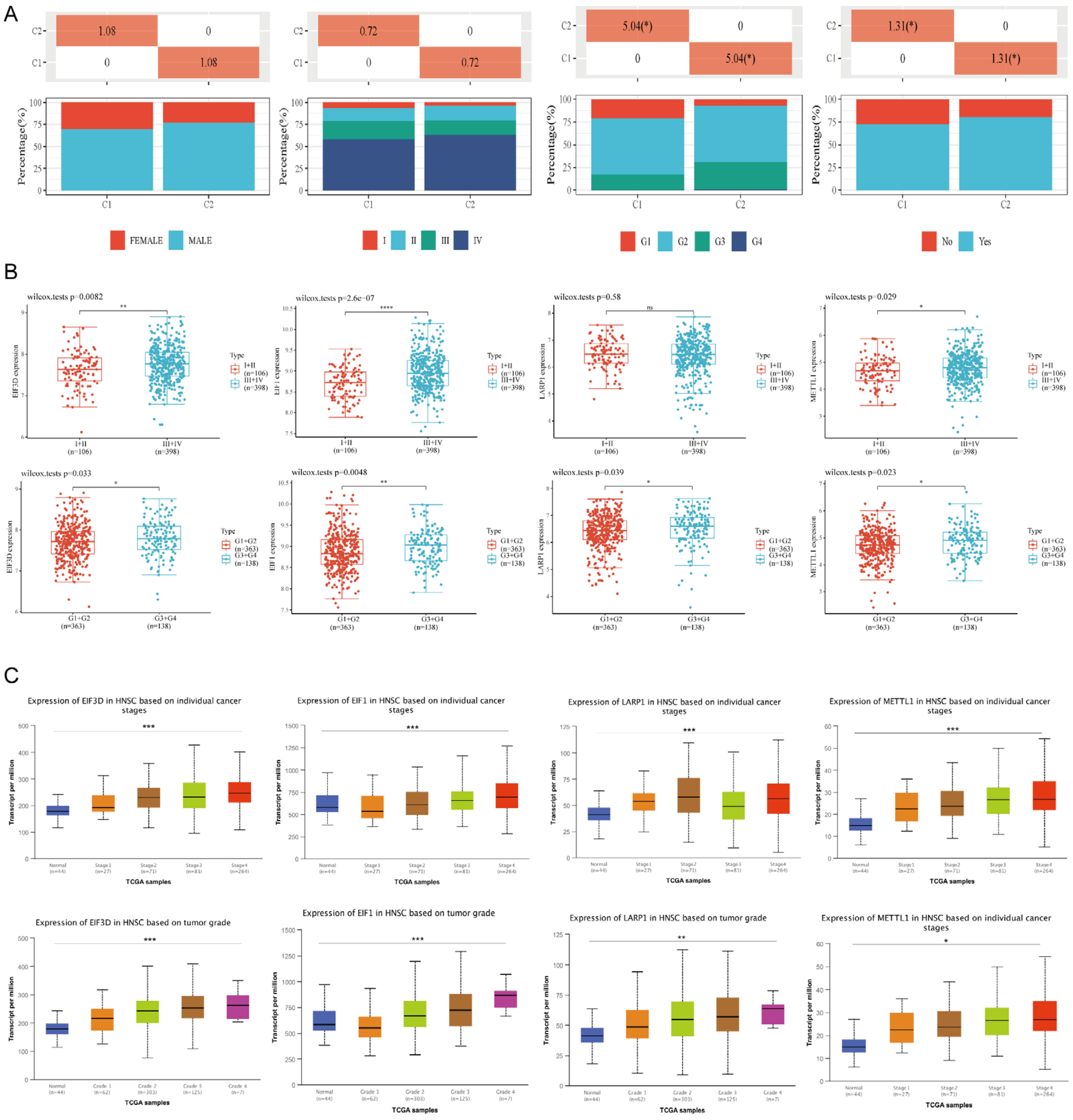

Figure S7 Correlation between m7GRGs and different clinicopathological characteristics of HNSCC. (A) Differences in clinicopathological features, including sex, clinical stage, grade, and smoking status, among the two subtypes of HNSCC; (B) Association of the four prognostic m7GRGs with tumor stage and HNSCC grade in the TCGA database; (C) Association of four prognostic m7GRGs with tumor stage and grade in the UALCAN database.

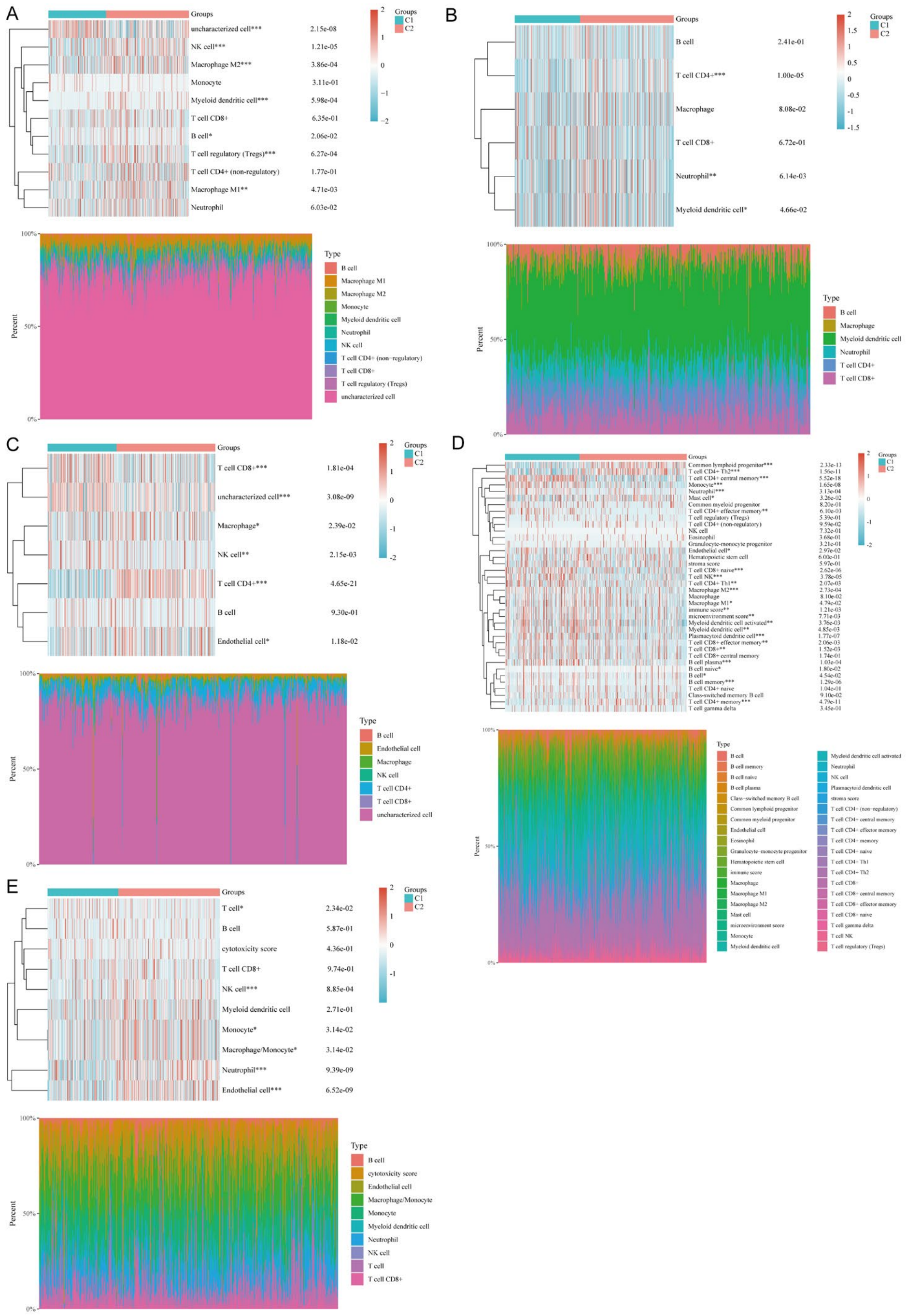

Figure S8. Relationship between the expression level of m7GRGs in the tumor microenvironment and immune infiltration (A-E) Comparison of immune scores between C1 and C2 subtypes in TCGA; (A) quantTIseq, (B) TIMER, (C) EPIC, (D) xCell, (E) MCPcounter.

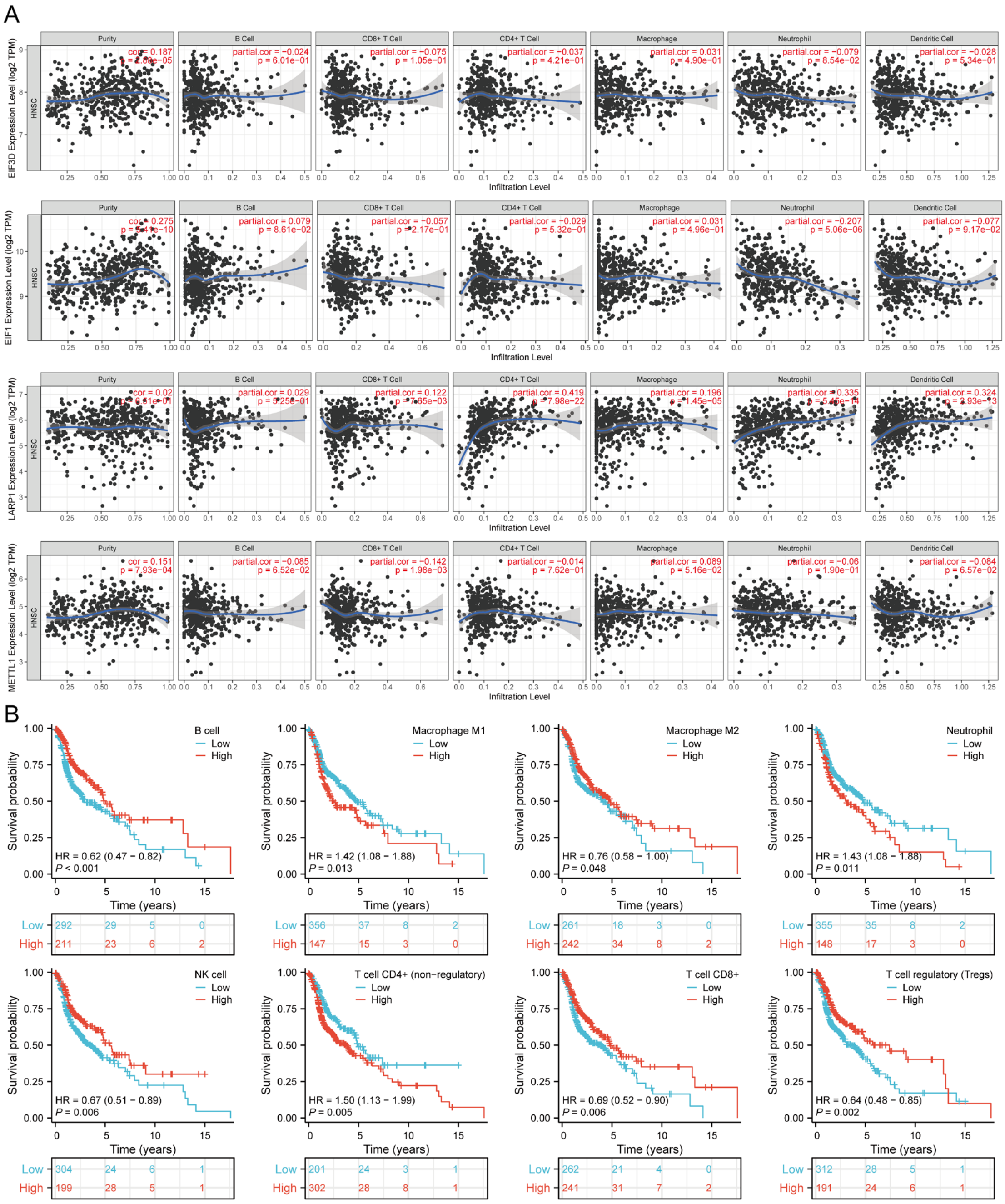

Figure S9. Relationship between the expression level of four prognostic m7GRGs and immune infiltration. (A) Relationship between the abundance of immune cells in HNSCC and the expression levels of four prognostic m7GRGs; (B) Survival analysis of different immune cell types.

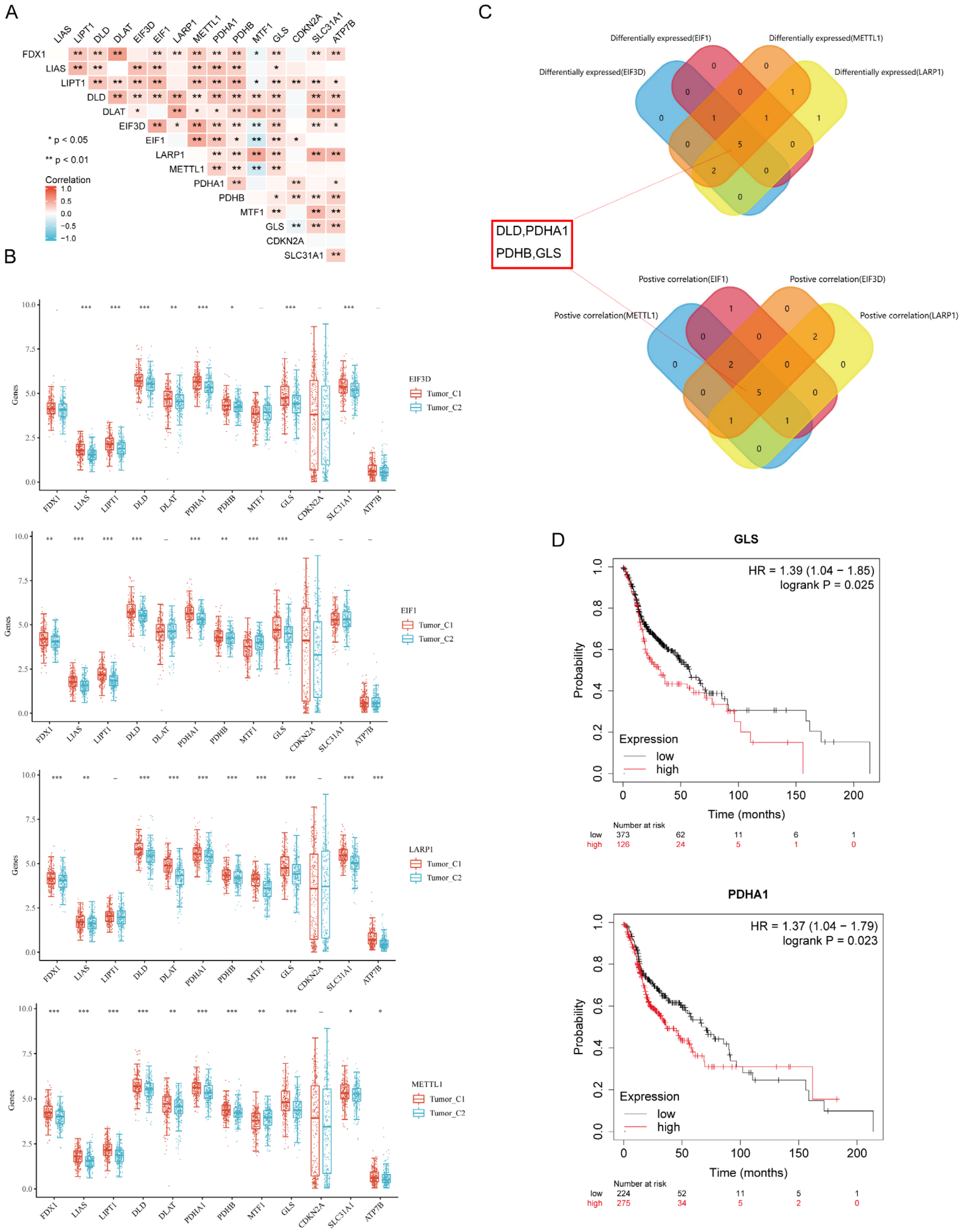

Figure S10. Correlation analysis between expression of four prognostic m7GRGs and CRGs in HNSCC. (A) Heat map showing correlation between expression of four prognostic m7GRGs and CRGs; (B) Differential expression of CRGs between high and low expression groups of four prognostic m7GRGs; (C) Venn diagram showing expression correlation and differential expression of CRGs, including PDHA1 and GLS; (D) Survival curve for PDHA1 and GLS.

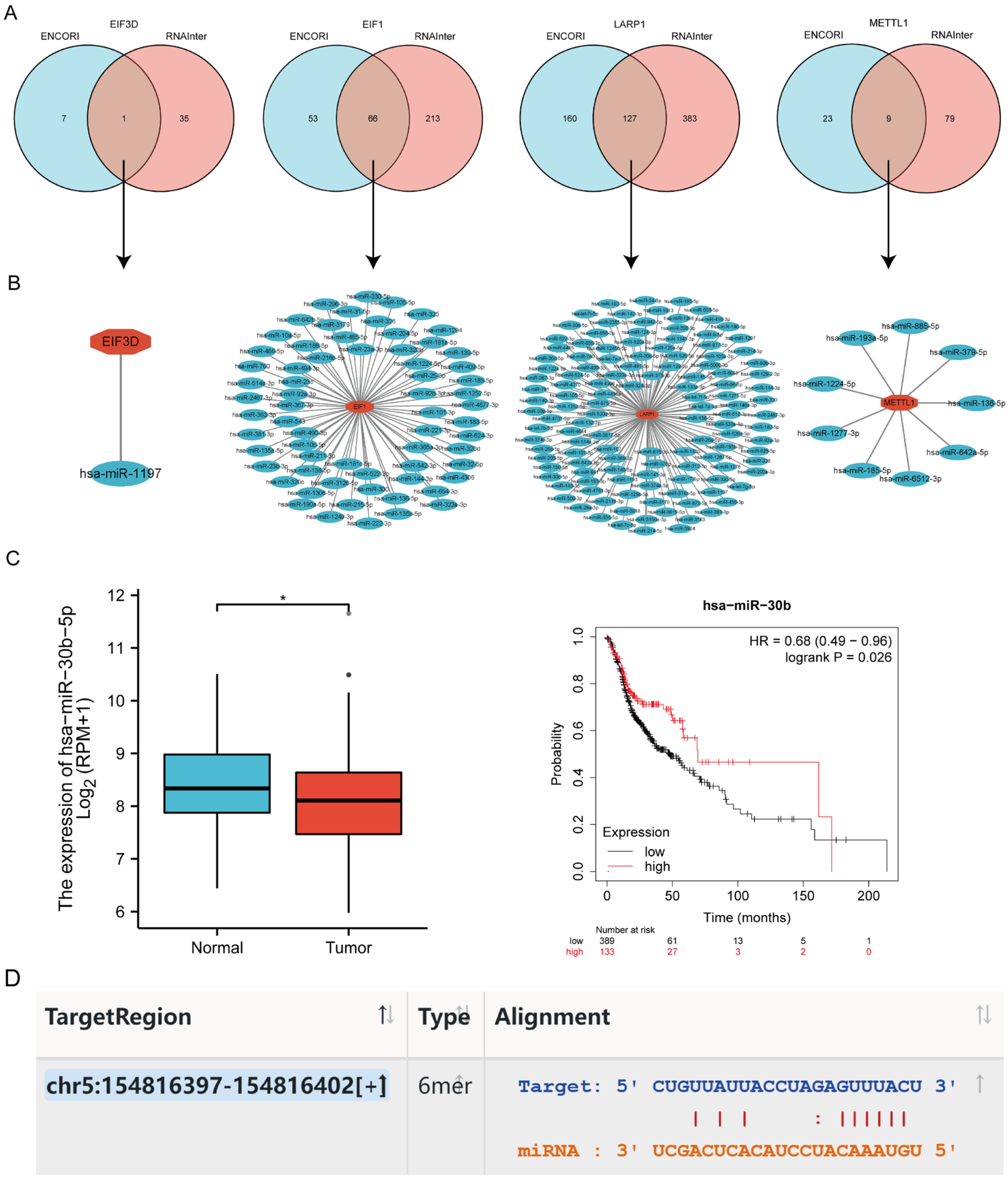

Figure S11. Identification of potential miRNAs associated with HNSCC prognosis. (A) Prediction of potential miRNAs of the four prognostic m7GRGs through ENCORI and RNAInter databases; (B) Construction of a potential miRNA gene network using Cytoscape software; (C) Expression and prognostic value of miRNAs (hsa-miR-30b-5p). (D) The 3'-UTR binding sites of potential miRNA to LARP1 were analyzed using Targetscan database.

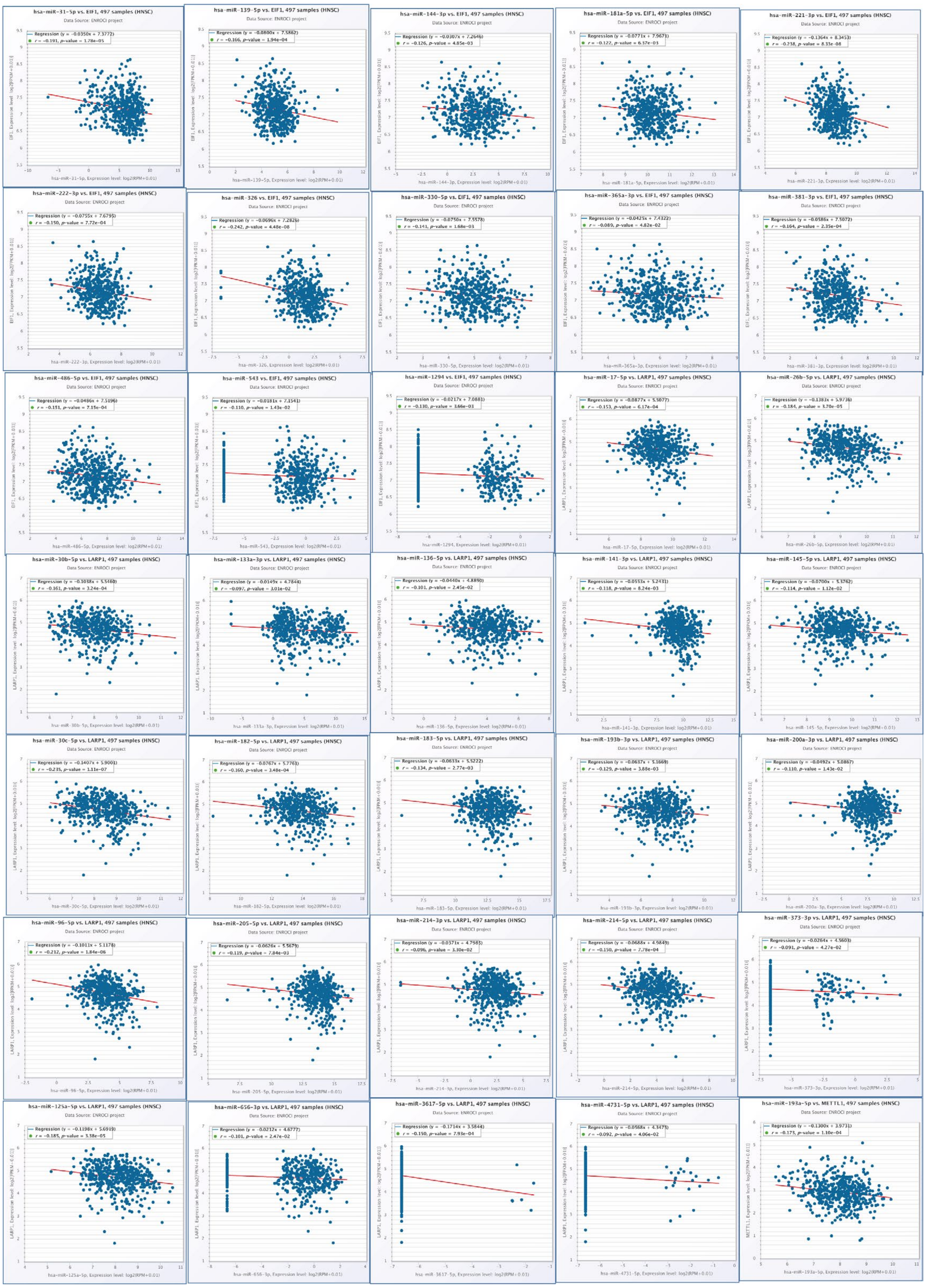

Figure S12. Correlation between potential miRNAs and m7GRG mRNA in HNSCC.

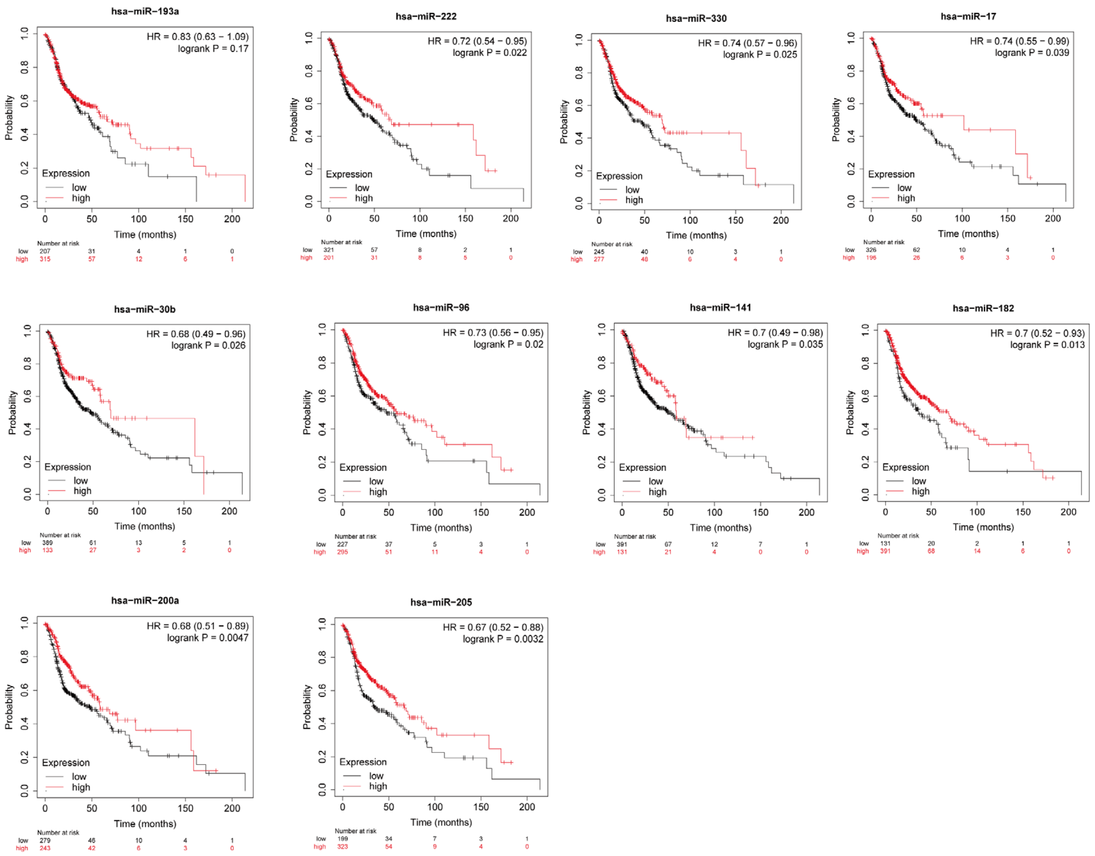

Figure S13. Prognostic evaluation of potential miRNAs in HNSCC.

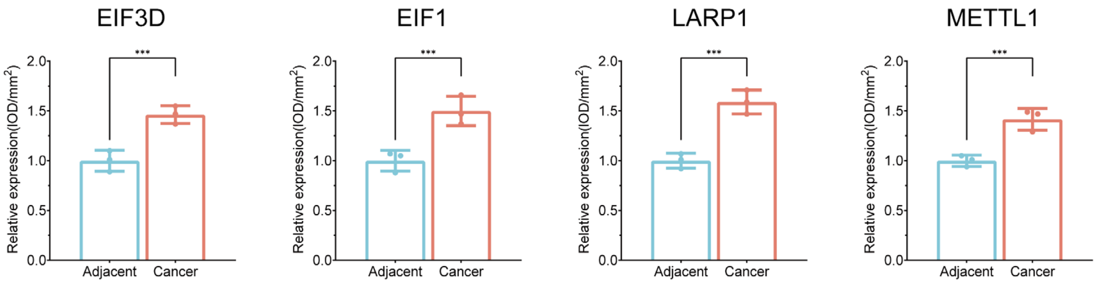

Figure S14. Relative expression levels of EIF3D, EIF1, LARP1, and METTL1 between HNSCC adjacent normal tissues and cancer tissues.
